# Diagnostic Stability of ICD/DSM First Episode Psychosis Diagnoses: Meta-analysis

**DOI:** 10.1093/schbul/sbw020

**Published:** 2016-03-15

**Authors:** Paolo Fusar-Poli, Marco Cappucciati, Grazia Rutigliano, Margaret Heslin, Daniel Stahl, Zera Brittenden, Edgardo Caverzasi, Philip McGuire, William T. Carpenter

**Affiliations:** ^1^Institute of Psychiatry Psychology Neuroscience, King’s College London, London, UK;; ^2^OASIS Clinic, SLaM NHS Foundation Trust, London, UK;; ^3^Department of Brain and Behavioural Sciences, University of Pavia, Pavia, Italy;; ^4^Department of Clinical and Experimental Medicine, University of Pisa, Pisa, Italy;; ^5^Maryland Psychiatric Research Center, University of Maryland School of Medicine, and VA Capitol Network (VISN 5) MIRECC, Baltimore, MD

**Keywords:** schizophrenia, diagnostic stability, psychosis, ICD, DSM

## Abstract

**Background:**

Validity of current International Classification of Disease/Diagnostic and Statistical Manual of Mental Disorders (ICD/DSM) first episode psychosis diagnoses is essential in clinical practice, research, training and public health.

**Method:**

We provide a meta-analytical estimate of prospective diagnostic stability and instability in ICD-10 or DSM-IV first episode diagnoses of functional psychoses. Independent extraction by multiple observers. Random effect meta-analysis conducted with the “metaprop,” “metaninf,” “metafunnel,” “metabias,” and “metareg” packages of STATA13.1. Moderators were tested with meta-regression analyses. Heterogeneity was assessed with the *I* ^2^ index. Sensitivity analyses tested robustness of results. Publication biases were assessed with funnel plots and Egger’s test.

**Findings:**

42 studies and 45 samples were included, for a total of 14 484 first episode patients and an average follow-up of 4.5 years. Prospective diagnostic stability ranked: schizophrenia 0.90 (95% CI 0.85–0.95), affective spectrum psychoses 0.84 (95% CI 0.79–0.89), schizoaffective disorder 0.72 (95% CI 0.61–0.73), substance-induced psychotic disorder 0.66 (95% CI 0.51–0.81), delusional disorder 0.59 (95% CI 0.47–0.71), acute and transient psychotic disorder/brief psychotic disorder 0.56 (95% CI 0.62–0.60), psychosis not otherwise specified 0.36 (95% CI 0.27–0.45, schizophreniform disorder 0.29 (95% CI 0.22–0.38). Diagnostic stability within schizophrenia spectrum psychoses was 0.93 (95% CI 0.89–0.97); changes to affective spectrum psychoses were 0.05 (95% CI 0.01–0.08). About 0.10 (95% CI 0.05–0.15) of affective spectrum psychoses changed to schizophrenia spectrum psychosis. Across the other psychotic diagnoses there was high diagnostic instability, mostly to schizophrenia.

**Interpretation:**

There is meta-analytical evidence for high prospective diagnostic stability in schizophrenia spectrum and affective spectrum psychoses, with no significant ICD/DSM differences. These results may inform the development of new treatment guidelines for early psychosis and impact drug licensing from regulatory agencies.

## Introduction

Unlike most of other areas of medicine, psychiatric diagnoses, as operationalized in the International taxonomies represented by the World Health Organization’s (International Classification of Diseases [ICD of WHO]) and American Psychiatric Association’s (Diagnostic and Statistical Manual of Mental Disorders [DSM of APA]), are derived from expert opinion,^1^ with the specific aim of improving reliability across clinicians. The National Institute of Mental Health commented: “unlike our definitions of ischemic heart disease, lymphoma or AIDS, the DSM diagnoses are based on a consensus about clusters of clinical symptoms, not any objective laboratory measure.”^2^ Since no objective tests or markers are on the horizon,^3^ clinical psychiatry is anchored to “the patient’s altered experience, expression and existence, associated with suffering in self and/or others.”^4^ Therefore, assessing validity of current diagnostic classification based on psychopathology^4^ is essential, in particular for patients at their first contact with mental health services.

The quantification of diagnostic stability and instability of first episode psychosis diagnoses is of paramount practical import,^5^ to ensure diagnostic validity^6^ and optimize early interventions,^7^ in light of the limited treatment achievements in the late stages of the disorder.^8,9^ For example, evidence of diagnostic stability within the schizophrenia spectrum psychoses and affective spectrum psychoses is fundamental to guide accurate early interventions. The National Institute of Health and Care Excellence (NICE) recommends the clinical guideline 178 (CG178) for schizophrenia spectrum psychoses, but CG38/90 in case of affective spectrum psychoses (recommendation 1.3.4.3^10^). These guidelines make substantive differential clinical recommendations such as use of pharmacological and psychological interventions, information and support for carers and patients, management of crisis and risk, long term care and strategies to promote recovery.^10^ Several studies addressing diagnostic stability of first episode psychosis diagnoses have been published, but the results are highly heterogeneous. For example, some studies suggested that the first episode schizoaffective disorder has the highest 2 years prospective diagnostic stability, followed by affective spectrum psychoses and only in third ranking by schizophrenia.^11^ Contrasting findings suggested that first episode schizophrenia exhibited the highest 2 years prospective diagnostic stability, and the schizoaffective disorder the lowest stability.^12^ Similarly, some studies found a greater stability for some first episode psychosis diagnoses as assigned using ICD-10 rather than DSM-IV criteria in the same patients,^11^ while others found the 2 systems to have similar prospective consistency.^13^

We present here the first meta-analysis of the diagnostic stability and instability of ICD-10/DSM-IV functional first episode psychosis diagnoses. Our first aim was to test the magnitude and consistency of prospective diagnostic stability of first episode psychosis diagnoses, while at the same time addressing diagnostic changes over time. Our analysis was complemented by investigation of some potential moderators (eg, ICD-10 vs DSM-IV). Our secondary aim was to meta-analytically address the magnitude of retrospective diagnostic stability of first episode psychosis diagnoses.

## Methods

### Search Strategy

Two independent investigators (M.C., Z.B.) conducted 2-step literature searches according to a specific protocol. First, the Web of Knowledge database was searched, incorporating both the Web of Science and MEDLINE. The search was extended until June 30, 2015, including abstracts in English language only. The electronic research adopted several combinations of the following keywords: “First episode psychosis,” “Diagnostic accuracy,” “Sensitivity,” “Specificity,” “Psychosis prediction,” “Psychosis onset,” “Diagnostic stability,” “Prediction,” “DSM-IV,” “ICD-10,” and “Follow-up”. Second, we used Scopus to investigate citations of possible previous reviews/meta-analyses on diagnostic stability of first episode psychosis diagnoses, and a manual search of the reference lists of retrieved articles. Articles identified through these 2 steps were then screened for the selection criteria on basis of abstract reading. The articles surviving this selection were assessed for eligibility on basis of full-text reading, following the MOOSE checklist (supplementary table 1).^14^

### Selection Criteria

Studies were eligible for inclusion if the following criteria were fulfilled: (a) were original articles, written in English; (b) included a group of ICD-10 or DSM-IV/DSM-IV-TR first episode psychosis patients (defined as first-ever admission to mental health services), diagnostically assessed at baseline and follow-up; and (c) reported the baseline and follow-up number of specific psychotic diagnoses: schizophrenia, schizoaffective disorders, schizophreniform disorder, affective spectrum psychoses, delusional disorder, substance-induced psychotic disorder, psychosis not otherwise specified, acute and transient psychotic disorder/brief psychotic disorder. Further details on the specific ICD-10/DSM-IV diagnostic codes are appended in supplementary methods. When data were not directly presented they were indirectly extracted from associated data. Corresponding authors were contacted to retrieve additional data when possible. Exclusion criteria were: (a) abstracts, pilot datasets, and papers in languages other than English; (b) articles not employing the internationally ICD/DSM validated diagnoses for psychosis; (c) articles not providing enough meta-analytical data; (d) articles reporting on organic psychoses, and (e) articles with overlapping datasets. Specifically, in case of multiple publications deriving from the same study population, we selected the articles reporting the largest and most recent data set. Literature search was summarized according to the PRISMA guidelines.^15^

### Recorded Variables

Data extraction was independently performed by 2 investigators (M.C., Z.B.). To estimate the primary outcome variable we extracted the baseline sample size and the number of patients with specific psychotic diagnoses at follow-up time. Moderators tested in meta-regression analyses are detailed below. Quality assessment is described below here.

### Quality Assessment

Quality assessment in observational research is controversial, with no clear consensus on rating methods or their appropriate use in the analysis. We adapted the Newcastle Ottawa Scale (NOS) for the evaluation of nonrandomized studies (http://www.ohri.ca/programs/clinical_epidemiology/oxford.asp). The scale evaluates the quality of observational studies allocating a maximum of 9 stars for higher quality. This tool has been adopted in recent meta-analyses.^16^

### Statistical Analysis

The primary outcome is the prospective diagnostic stability/instability of ICD/DSM first episode psychosis diagnoses over time. The prospective diagnostic stability is defined as the proportion of baseline patients retaining the same psychotic diagnosis over time.^12^ In case of remission or full recovery at follow-up, the initial diagnosis is unchanged. The prospective diagnostic instability of first episode psychosis diagnoses over time is defined as the complementary proportion of baseline patients shifting to other diagnoses at follow-up. The prospective diagnostic stability/instability was computed across each initial diagnostic category. We performed additional analyses clustering the individual diagnoses across diagnostic spectra relevant for the NICE clinical guidelines^10^: schizophrenia spectrum psychoses (schizophrenia, schizophreniform disorder, schizoaffective disorder) and affective spectrum psychoses (mania with psychosis and/or bipolar disorder with psychosis and/or depression with psychosis). The secondary outcome is the retrospective diagnostic stability/instability across each diagnosis, defined as the proportion of follow-up patients that receives the same diagnosis that they had at baseline, or another diagnosis as compared to that received at baseline respectively.^12^ Meta-analysis was conducted with the “metaprop” package^17^ of Stata 13.1. This package is specifically developed for pooling proportions in a meta-analysis of multiple studies. The CIs are based on score (Wilson) procedures.^18^ As proportions were expected to be often small we used Freeman-Tukey Double Arcsine transformation^19^ to stabilize the variances and then we performed a random effect meta-analysis implementing the Der Simonian-Laird method.^20^ The influence of moderators (age, gender, time to follow-up, comorbid substance abuse, ICD-10 vs DSM-IV diagnostic criteria, publication year, quality assessment, clinical setting of initial diagnosis, baseline functional level measured with the Global Assessment of Functioning [GAF] scale, duration of untreated psychosis) on the diagnostic instability of each diagnostic category was tested using meta-regression analyses with the “metareg” function.^21^ The slope of meta-regression line (β-coefficient: direct [+] or inverse [−]) indicates the strength of a relationship between moderator and outcome. The meta-regressions were conducted when at least 10 studies were available for each moderator.^22^ Because of the large number of regressions the alpha level was reduced to .01^23^ as a compromise between a strict control of the familywise error and not having any power to detect any relationships. Tests with a *P* value between .01 and .05 were discussed as trends. Subgroup analyses were additionally used to further investigate the impact of ICD-10 vs DSM-IV criteria on diagnostic stability and instability of each specific psychotic diagnosis. Heterogeneity among study point estimates was assessed using Q statistics with the proportion of the total variability in the effect size estimates being evaluated with the *I* ^2^ index,^24^ which does not depend upon the number of studies included. As meta-analysis of observational studies is supposed to be characterized by significant heterogeneity, random effect models were used. Sensitivity analyses were also conducted to investigate the influence of each single study on the overall prospective diagnostic instability by omitting 1 study at a time, using Stata’s user-written function, “metaninf.”^25,26^ A study was considered to be influential if the pooled mean estimate without it was not within the 95% confidence bounds of the overall mean. Publication biases were assessed with the “metafunnel” function of Stata which produced funnel plots for assessing small-study reporting bias in meta-analysis^27^ and with the “metatrim”^28^ function of Stata.

## Results

### Database

**Fig. 1.**
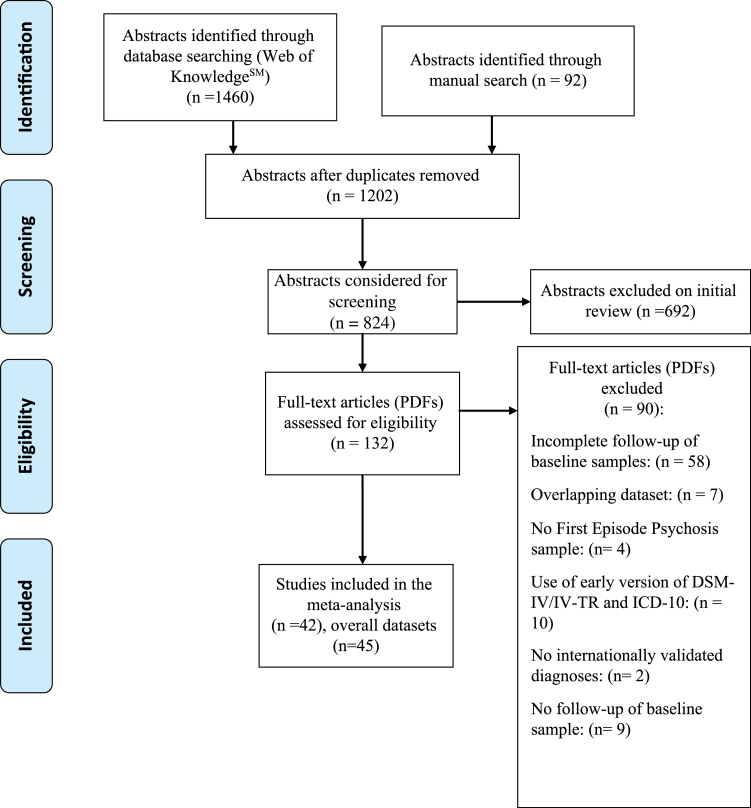
PRISMA Flow Chart.

Literature search (PRISMA flow-chart figure 1) uncovered 42 independent articles. The list of excluded studies is detailed in the supplementary table 2. There were 23 studies employing ICD-10 and 22 employing DSM-IV, with 3 studies contributing 2 samples (ICD-10 and DSM-IV) each,^13,29,30^ for an overall of 45 independent samples. The final database comprised 14 484 first episode patients, 10 510 diagnosed with ICD-10 and 3974 diagnosed with DSM-IV. The mean age of the patients was 29 years (median 27 years, age range 16–75) and the mean proportion of females was 0.49. Age, gender, diagnostic instrument employed to assign the psychotic diagnosis, quality assessment, baseline sample size are detailed in table 1, while duration of untreated psychosis and operationalization of first episode of psychosis are detailed in supplementary table 3. The mean follow-up time was 4.5 years (*n* = 45, 53.86 months, SD = 51.51, IQR 23–77). All studies but one^31^ employed the same diagnostic instrument at baseline and follow up.

**Table 1.** List of Studies Included in the Meta-analysis

Study Name and Year of Publication	NOS Score (9 = Max.); Comorbid Substance Misuse (%)	Diagnostic Manual	FEP Whole Sample	Age (Mean ± SD)	Gender (% Females)	Follow-up (Months ± SD)
1. Okasha, et al 1993^32^	5; NA	ICD-10	50	27±9.8	50	12 ± NA
2. Jørgensen, et al 1995^33^	5; NA	ICD-10	75	43±14	60	96 ± NA
3. Jørgensen, et al 1997^34^	6; NA	ICD-10	46	NA	NA	12 ± NA
4. Das, et al 1999^35^	4; No	ICD-10	40	25.7±9	70	1 ± NA
5. Schwartz, et al^a^ 2000^12^	8; Yes (30.2)	DSM-IV	514^b^	28 ± NA	NA	24 ± NA
6. Sajith, et al 2002^36^	6; NA	ICD-10	45	26.9±10.9	71.1	36.5 ± NA
7. Jäger, et al 2003^31^	5; NA	ICD-10	73	31.8±14.6	50.7	60 ± NA
8. Jarbin, et al 2003^37^	6; Yes (NA)	DSM-IV	67^b^	16 ± NA	51.5	122.4 ± NA
9. Amini, et al 2005^29^	6; Yes (NA)	DSM-IV; ICD-10	48	23.5±7.7	45.8	12 ± NA
10. Correll, et al 2005^38^	5; Yes (NA)	DSM-IV	26	16.2±2.7	34.5	22.8±19.8
11. Rufino, et al 2005^39^	4; No	DSM-IV	59	NA	27.1	19.4±6.1
12. Schimmelmann, et al 2005^40^	6; Yes (59.1)	DSM-IV	492	22±3.6	36.8	18 ± NA
13. Suda, et al 2005^41^	6; NA	ICD-10	25	37.8±9.5	76	116.4±45.6
14. Whitty, et al 2005^42^	6; Yes (NA)	DSM-IV	147	NA	NA	48 ± NA
15. Abe, et al 2006^43^	5; NA	ICD-10	16	34.8±9.9	50	144 ± NA
16. Addington, et al 2006^44^	7; Yes (43)	DSM-IV	228	24.5±8.2	32.9	12 ± NA
17. Thangadurai, et al 2006^45^	4; NA	ICD-10	87	29.8±11	48	13.2±11.7
18. Boks, et al 2006^46^	5; NA	DSM-IV	29	26.9±6.3	37.9	24 ± NA
19. Rahm, et al 2007^47^	6; Yes (NA)	DSM-IV	146	NA	NA	36 ± NA
20. Subramaniam, et al 2007^48^	6; No	DSM-IV	154	28.4±6.6	49	24 ± NA
21. Fraguas, et al 2008^49^	4; No	DSM-IV	24	15.7±1.6	25	24
22. Haahr, et al 2008^50^	6; Yes (38)	DSM-IV	279^b^	NA	NA	24 ± NA
23. Chang, et al 2009^51^	6; Yes (11.7)	ICD-10	166	19.8±3.1	46.4	53.4±13.3
24. Crebbin, et al 2009^52^	6; Yes (100)	ICD-10	35	25.6±8.4	17.1	24 ± NA
25. Pedrós, et al 2009^53^	7; Yes (NA)	DSM-IV	48	28.1±8.7	NA	24 ± NA
26. Salem, et al 2009^54^	5; NA	ICD-10	69	27.5±6.6	32.4	72 ± NA
27. Salvatore, et al 2009^55^	7; Yes (51.2)	DSM-IV-TR	500	31.7±13.7	45	24 ± NA
28. Aadamsoo, et al 2011^56^	6; NA	ICD-10	107	NA	60	24 ± NA
29. Barak, et al 2011^57^	5; Yes (NA)	ICD-10	83	75.4±9.3	56.6	27.7 ± NA
30. Castro-Fornieles, et al 2011^58^	7; Yes (NA)	DSM-IV	83	15.5±1.7	32.5	24 ± NA
31. Kim, et al 2011^59^	6; No	DSM-IV	150	27.7±9.5	NA	27.1±25.3
32. Möller, et al 2011^30^	8; NA	DSM-IV; ICD-10	136; 123	NA	NA	180 ± NA
33. Salvatore, et al 2011^11^	8; Yes (51.2)	ICD-10	500	31.7±13.7	45	24 ± NA
34. Narayanaswamy, et al 2012^60^	5; Yes (8.8)	ICD-10	57	30.7±11.8	65	24 ± NA
35. Pillmann, et al 2012^61^	7; NA	ICD-10	71	NA	NA	148.8±87.6
36. Kingston, et al^a^ 2013^62^	8; No	DSM-IV	187^b^	42.7±18.6	41.3	76.8±27.6
37. Pope, et al 2013^63^	6; Yes (31.3)	DSM-IV-TR	214	22.6±4	30.4	12 ± NA
38. Castagnini & Foldager 2014^64^	8; Yes (NA)	ICD-10	5426	28±11.7	47.4	111.6
39. Kapfhammer, et al 2014^65^	6; No	DSM-IV-TR	55^b^	NA	NA	144 ± NA
40. Queirazza, et al 2014^66^	7; Yes (NA)	ICD-10	2923	37.4±17.7	45.5	48±47.6
41. Heslin, et al 2015^13^	8; Yes (NA)	DSM-IV-TR; ICD-10	402^b^; 401^b^	30.8±10.7	42.9	128.9±14
42. Ranjan, et al 2015^67^	5; NA	ICD-10	30	NA	NA	3 ± NA

*Note*: DSM-IV/IV-TR, Diagnostic and Statistical Manual of Mental Disorders, 4th Edition/Text Revision; ICD-10; International Statistical Classification of Diseases and Related Health Problems, 10th Edition; FEP, First Episode Psychosis; NA, data not available; NOS, Newcastle Ottawa Scale.

^a^Extended baseline assessment.

^b^After excluding patients not meeting inclusion criteria (ie, with organic psychoses or with non-psychotic diagnoses).

### Meta-analytical Prospective Diagnostic Stability of ICD/DSM First Episode Psychosis Diagnoses

Meta-analytical diagnostic stability of first episode psychosis diagnoses across each diagnostic category is detailed in the boldfaced diagonal of table 2, while diagnostic instability is detailed in the other cells. Between group analyses found no ICD-10 vs DSM-IV significant differences.

**Table 2.** Meta-analytical Cross Tabulation of Prospective Stability and Instability (Average 4.5 y) of DSM-IV/ICD10 First Episode Psychosis Diagnoses

		Follow-up Diagnosis																	
		SZ		SA		SF (DSM Only)		ASP		DD		ATPD/BPD		SI		PNOS		Others	
Baseline Diagnosis	Studies, Subjects	Mean	95% CI	Mean	95% CI	Mean	95% CI	Mean	95% CI	Mean	95% CI	Mean	95% CI	Mean	95% CI	Mean	95% CI	Mean	95% CI
SZ (ICD/DSM)	24, 1603	**0.9**	**0.85–0.95**	0.04	0–0.09	0	0–0.06	0.03	0–0.08	0	0–0.05	0	0–0.05	0.03	0–0.08	0	0–0.06	0	0–0.05
SA (ICD/DSM)	19, 317	0.16	0.05–0.27	**0.72**	**0.61–0.83**	0.01	0–0.14	0.08	0–0.19	0	0–0.1	0	0–0.11	0.01	0–0.12	0.02	0- 0.12	0.01	0–0.12
SF (DSM only)	20, 573	0.57	0.48–0.65	0.05	0–0.12	**0.29**	**0.22–0.38**	0.06	0,01–0,08	0	0–0.08	0.01	0–0.4	0.01	0–0.11	0.01	0–0.08	0	0–0.08
ASP (ICD/DSM)	23, 1705	0.05	0–0.09	0.05	0.01–0.1	0	0–0.06	**0.84**	**0.79–0.89**	0.01	0–0.05	0	0–0.05	0.01	0–0.05	0.01	0–0.06	0.03	0 -0.07
DD (ICD/ DSM)	22, 253	0.29	0.17–0.41	0.04	0–0.16	0.01	0–0.16	0.05	0–0.17	**0.59**	**0.47–0.71**	0	0–0.13	0.01	0–0.13	0.01	0–0.14	0	0–0.13
ATPD/BPD (ICD, DSM)	40, 9412	0.21	0.16–0.25	0.02	0–0.06	0.02	0–0.14	0.12	0.07–0.16	0.01	0–0.06	**0.56**	**0.52–0.60**	0.01	0–0.05	0.01	0–0.06	0.04	0–0.09
SI (ICD/DSM)	10, 164	0.17	0.1–0.31	0.04	0–0.19	0.02	0–0.21	0.06	0–0.21	0.02	0–0.17	0.02	0–0.18	**0.66**	**0.51–0.81**	0.01	0–0.17	0	0–0.16
PNOS (ICD/ DSM)	24, 457	0.31	0.21–0.39	0.1	0.01–0.19	0.02	0–0.12	0.07	0–0.16	0.03	0–0.12	0.04	0–0.14	0.01	0–0.10	**0.36**	**0.27–0.45**	0.06	0–0.16

*Note*: SZ, schizophrenia; SA, schizoaffective disorder; SF, schizophreniform disorder; ASP, affective spectrum psychoses; DD, delusional disorder; ATPD/BPD, acute and transient psychotic disorder/brief psychotic disorder; SI, substance-induced psychotic disorder; PNOS, psychosis not otherwise specified; Other, mental disorder other than psychosis. In all the cells, subgroup analyses (ICD-10 vs DSM-IV) revealed no significant between groups differences. Diagnostic stability and instability results stratified for ICD-10 and DSM-IV are appended in supplementary materials. The diagnostic stability data are **boldfaced**.

Point estimate diagnostic stability of first episode psychosis diagnoses was highest for schizophrenia (0.90), and high for affective spectrum psychoses (0.84). Diagnostic stability was moderate for schizoaffective disorder (0.72) and moderate to low for substance-induced psychotic disorder (0.66), delusional disorder (0.59) and acute psychotic disorder/ brief psychotic disorder (0.56). Diagnostic stability was very low for psychosis not otherwise specified (0.36) and schizophreniform disorder (0.29). In a subset of studies we further addressed the diagnostic stability of mania/bipolar disorder with psychosis (0.90) and depression with psychosis (0.73) separately (supplementary table 4).

When the analyses were repeated across the schizophrenia vs affective psychosis spectra (table 3), diagnostic stability of schizophrenia spectrum psychoses was 0.93, while for affective spectrum psychoses it was 0.84. Changes from a schizophrenia spectrum to an affective spectrum were infrequent, accounting for only 0.05 of initial cases. On the other hand, about 0.1 of initial affective spectrum psychoses shifted towards schizophrenia spectrum psychoses.

**Table 3.** Meta-analytical Cross Tabulation of Prospective Diagnostic Stability and Instability (Average 4.5 y) of ICD-10/DSM-IV First Episode Schizophrenia Spectrum and Affective Spectrum Psychoses

		Follow-up Diagnosis													
Baseline Diagnosis		Schizophrenia Spectrum		Affective Spectrum		DD		ATPD/BPD		SI		PNOS		Other	
	Studies, Subjects	Mean	95% CI	Mean	95% CI	Mean	95% CI	Mean	95% CI	Mean	95% CI	Mean	95% CI	Mean	95% CI
Schizophrenia spectrum psychoses (ICD-10/ DSM-IV)	26, 2493	**0.93** ^**a**^	**0.89–0.97**	0.05	0.01–0.08	0	0–0.04	0	0–0.04	0.01	0–0.05	0.01	0–0.05	0	0–0.04
Affective spectrum psychoses (ICD-10/ DSM-IV)	23, 1705	0.1	0.05–0.15	**0.84** ^**b**^	**0.79–0.89**	0.01	0–0.05	0	0–0.05	0.01	0–0.06	0.01	0–0.06	0.03	0–0.08

*Note*: DD, delusional disorder; ATPD/BPD, acute and transient psychotic disorder/brief psychotic episode; SI, substance-induced psychotic disorder; PNOS, psychosis not otherwise specified; Other, mental disorder other than psychosis. Schizophrenia spectrum psychoses: schizoaffective disorder, schizophreniform disorder, schizophrenia. The diagnostic consistency data are **boldfaced**.

^a^Subgroup analyses within the studies enrolling first episode patients aged less than 16 years old with available data^37,49,58^: mean 0.95, 95% CI 0.84–1.

^b^Subgroup analyses within the studies enrolling first episode patients aged less than 16 years old with available data^37,49,58^: mean 0.84, 95% CI 0.73–0.93.

About one-third of the initial cases of delusional disorders shifted towards schizophrenia spectrum disorders (0.29 schizophrenia, 0.04 schizoaffective disorder). Diagnostic changes were also frequent from an initial acute and transient psychotic disorder/brief psychotic disorder: one-fourth of them towards schizophrenia spectrum disorders (0.21 schizophrenia, 0.02 schizophreniform disorder, 0.02 schizoaffective disorder) and 0.12 of them towards affective spectrum psychoses. Substance-induced psychotic disorder shifted most frequently towards schizophrenia spectrum psychoses (0.17 schizophrenia, 0.04 schizoaffective disorder, 0.02 schizophreniform disorder). Among the initial cases of psychosis not otherwise specified, about one-third (0.36) retained the initial diagnosis and one-third (0.31) shifted towards schizophrenia.

### Meta-regressions, Publication Biases, and Sensitivity Analyses

Meta-regressions analyses across each psychotic category clarified that the publication year impacted diagnostic instability of schizophrenia, schizophreniform disorder, delusional disorder (at trend level uncorrected for multiple comparisons) and acute and transient psychotic disorder/brief psychotic disorder (corrected for multiple comparisons, supplementary table 5), with higher instability over the most recent years. Similarly, the inpatient setting during formulation of an initial diagnosis was associated with reduced diagnostic instability as compared to a mixed setting for schizophrenia, affective spectrum psychoses, delusional disorder (at trend level uncorrected for multiple comparisons), acute and transient psychotic disorder/brief psychotic disorder (corrected for multiple comparisons, supplementary table 5). A better quality of studies was associated with increased diagnostic instability for acute and transient psychotic disorder/brief psychotic disorder (corrected for multiple comparisons, supplementary table 5). There was no effect for age, gender, comorbid substance abuse, baseline ICD-10 or DSM-IV diagnostic criteria, baseline GAF level and duration of follow-up while there were not enough studies to investigate the effect of duration of untreated psychosis (supplementary table 5).

Visual inspection of funnel plots revealed no asymmetry, and metatrim analyses identified no studies to cut and fill (supplementary figure 1). Sensitivity analyses of overall diagnostic instability of ICD-10 (supplementary figure 2a) and DSM-IV (supplementary figure 2b) studies uncovered no outliers and confirmed robustness of results.

### Meta-analytical Retrospective Diagnostic Stability of ICD/DSM First Episode Psychosis Diagnoses

Meta-analytical retrospective diagnostic stability was high for acute and transient psychotic disorder/brief psychotic disorder and schizophreniform disorder, modest for affective spectrum psychoses and delusional disorder, low for schizophrenia and schizoaffective disorder, psychosis not otherwise specified and substance-induced psychotic disorder (supplementary table 6), averaging 0.6 for schizophrenia spectrum psychoses and 0.64 for affective spectrum psychoses (supplementary table 7).

## Discussion

We present here the first meta-analytical estimate of diagnostic stability of first episode psychosis diagnoses. Our database was large, including 42 studies and 45 samples for a total of 14 484 subjects, followed up for a mean period of 4.5 years. There was significant construct variability^68^ across prospective diagnostic stability of different first-episode psychotic diagnoses: schizophrenia 0.90, affective spectrum psychoses 0.84, schizoaffective disorder 0.72, substance-induced psychotic disorder 0.66, delusional disorder 0.59, acute and transient psychotic disorder/brief psychotic disorder 0.56, psychosis not otherwise specified 0.36, and schizophreniform disorder 0.29.

This is the first robust meta-analytical evidence documenting a very high diagnostic stability of schizophrenia, which is comparable to other clinical diagnoses in medicine. For example, a study in 4141 patients affected with dementia or mild cognitive impairment reported a similar (2 y) prospective diagnostic stability of 0.91.^69^ Another study in 783 patients affected with migraine or tension type headache reported a (7 mo) diagnostic stability of 0.66 and 0.63, respectively.^70^ Prospective stability of specific first-episode diagnoses may be of relevance for agencies licensing the use of drugs and medical devices such as the Food and Drug Administration (FDA) in the United States or the Medicines & Healthcare Products Regulatory Agency (MHRA) in United Kingdom and the European Medicine Agency (EMA) in the European Union. For example, the FDA has approved the use of paliperidone for the treatment of schizophrenia and schizoaffective disorder only, risperidone for schizophrenia and bipolar disorder (and irritability associated with autism), fluphenazine for psychotic disorders in general.^71^ Clozapine is licensed only for treatment resistant or suicidal risk schizophrenia.^71^ The off-license use of these medications for psychotic diagnoses is common^72^ and may have legal or health economic implications.

On a clinical level, not all aspects of diagnostic instability are negative. Although evidence of diagnostic stability is one of the key criteria for establishing the validity of most first episode psychosis diagnoses,^6^ some nosologic categories (eg, schizophreniform disorder or psychosis not otherwise specified) are formulated a priori on expected diagnostic uncertainty at the onset of psychosis or inadequate information available for specific diagnosis. Such diagnostic categories are intended as “place-holders.” Frequent diagnostic shifts in these disorders are to be expected. Interestingly, we found that about one-third of initial cases of schizophreniform disorder or psychosis not otherwise specified retained their initial diagnosis, suggesting some ongoing clinical uncertainty or clinician reluctance to specify a category. To overcome these issues and better understand the clinical relevance of our findings, we reported a high diagnostic stability across schizophrenia spectrum psychoses (0.93), as well as across affective spectrum psychoses (0.84). Changes from schizophrenia spectrum to affective spectrum were infrequent (0.05), and about 0.1 of the initial affective spectrum psychoses shifted towards schizophrenia spectrum psychoses. These findings may be of direct clinical relevance to clinicians who are required to follow the differential NICE guidelines for early schizophrenia spectrum vs affective spectrum psychoses. The concern that initial first-episode diagnosis, if incorrect, may impede clinical care is particularly relevant for changes between schizophrenic and affective spectra and less so within the same spectrum. Differences between an initial diagnosis of major depressive disorder with psychotic features and that of schizophrenia are profound, not only in the pharmacotherapies and specific forms of psychological therapy typically used “but also in the descriptions provided to newly diagnosed individuals and their families as to what lies ahead.”^5^ Indeed, recent epidemiological studies in first episode samples have confirmed that schizophrenia spectrum diagnoses have a worse clinical, social and service use course and outcome as compared to affective spectrum diagnoses.^73^

The additional clinically relevant finding of our meta-analysis is of no significant differences across ICD-10 vs DSM-IV definitions of each first episode diagnosis, as revealed by meta-regression analyses. Such a result may well reflect the increasing effort of international diagnostic manuals to improve reliability of psychiatric diagnoses and to harmonize the ICD and DSM.^74^ Our results may also be used to inform future evaluations and revisions of the new ICD-11 or DSM-5 diagnostic criteria. For example, given existing concerns that schizoaffective disorder may be a heterogeneous pathology in terms of the longitudinal course,^75^ we confirmed a good diagnostic stability (0.72). We also found that about 0.16 initial cases of ICD-10/DSM-IV schizoaffective disorder would shift towards schizophrenia. This value can be used as reference, to test if the new DSM-5 diagnostic criteria for schizoaffective disorder^76^ have actually been successful at improving diagnostic reliability by shifting the concept from episode to life course of the disorder.

We also showed that first episode psychosis diagnoses other than schizophrenia or affective spectrum psychoses had a low diagnostic stability. Diagnostic changes were frequently to schizophrenia: 0.31 of initial psychosis not otherwise specified, 0.29 of initial delusional disorders, 0.21 of initial acute and transient psychotic disorder/brief psychotic disorder, and 0.17 of initial substance-induced psychotic disorder. Because of these changes to schizophrenia, the retrospective diagnostic stability of schizophrenia spectrum disorders was low, suggesting that a significant number of the patients may be misdiagnosed at baseline. Careful monitoring and reassessment of patients presenting with unstable and remitting first episode diagnoses, such as acute and transient psychotic disorder/brief psychotic disorder,^77^ seems especially important.

Specific predictors of diagnostic instability need to be determined in order to identify patients who may be misdiagnosed at baseline. We could not identify significant meta-analytical variance in terms of age, gender, ICD-10 vs DSM-IV criteria, baseline GAF levels, duration of follow-up and comorbid substance abuse. We did not restrict first episode samples to a specific age range (eg, 16–35) to follow the new international guidelines that have eliminated young age as eligibility criterion to access early intervention services.^78^ The NICE CG 1.3.1.1 recommends that “early intervention services should be accessible to all people with a first episode or first presentation of psychosis, irrespective of the person’s age or the duration of untreated psychosis”. Variability of age across the samples included in the current analysis allowed investigating its impact on meta-analytical outcomes. For example, the median age of our sample was of 27 years, with a few outliers in the lower^58^ and upper range.^57^ However, the outliers had no impact on the meta-analytical estimates, as shown by the sensitivity analyses (supplementary figure 2a and supplementary figure 2b). Furthermore, no significant change in diagnostic stability was observed when the meta-analysis was repeated in the subset of studies enrolling first episode patients aged 16 (footnotes a and b to table 3). Overall, the median age of our sample is consistent with evidence indicating that prodromal phase typically starts at 21 years and that it may last a few years.^79^ Another significant limitation of the current analysis is that we could not assess the impact of duration of untreated psychosis. Therefore the delay from the onset of symptoms to the diagnostic assessment is undetermined. Meta-analyses carry over limitations in the original studies and there were not enough studies reporting on these moderators. Similarly we cannot exclude that other confounders not assessed by our meta-regressions such as quality of the diagnostic assessment may have a significant impact on the diagnostic stability. However, we did assess the overall methodological quality of the included studies with the NOS^16^ and we found no significant effects for most ICD/DSM diagnoses, with the exception of ATPD/BPD. The lack of any effect of comorbid substance use may be due to the fact that different substances may have opposite effects on diagnostic stability. There is evidence for cannabis, but not stimulants, being associated with diagnostic instability.^80^ With respect to duration of follow-up, the median follow-up time of the included studies was 2 years, therefore it is probable that most diagnostic changes would have already occurred by this time. Diagnostic re-assessment of first episode cases early in the course of illness seems indicated. The lack of impact of baseline functional level on diagnostic stability supports the recent decision of the DSM-5 Task Force to eliminate the GAF, an inadequate instrument for assessment of psychiatric functional impairment.^81^ Conversely, we found greater diagnostic stability when the initial diagnosis was formulated in an inpatient unit as compared to a mixed clinical setting. Specifically, our meta-analysis suggests that diagnostic stability of schizophrenia, affective spectrum psychoses, delusional disorder and acute and transient psychotic disorder/brief psychotic disorder could be significantly improved when the initial diagnosis is made in an inpatient unit. This finding supports an earlier report^1^ and suggests that more severe psychopathology and/or more continuous observation maximizes stability of initial diagnosis.

## Conclusions

There is meta-analytical evidence for high prospective diagnostic stability in schizophrenia spectrum psychoses followed by affective spectrum psychoses, with no significant ICD/DSM differences. Diagnostic stability across the other first episode psychotic diagnoses was low and most of diagnostic changes were to schizophrenia. Diagnostic stability is important to patients and caretakers and provides general guidance for clinical decision making. Stability is important for regulatory purposes and development of treatment guidelines. Addressing instability of diagnosis is an important challenge for future diagnostic development of early psychosis.

## Supplementary Material

Supplementary material is available at http://schizophreniabulletin.oxfordjournals.org.

## Funding

This study was supported in part by a 2014 NARSAD Young Investigator Award to P.F-P. D.S. was supported in part funded by the National Institute for Health Research (NIHR) Biomedical Research Centre at South London and Maudsley National Health System (NHS) Foundation Trust and King’s College London. The views expressed are those of the author(s) and not necessarily those of the NIHR.

## Supplementary Material

Supplementary Data
