# Supplementary material for: Diagnostic Stability of ICD/DSM First Episode Psychosis Diagnoses: Meta-analysis
Source: Schizophr Bull. 2016 Mar 15;42(6):1395–406. doi: 10.1093/schbul/sbw020 (PMC5049518; doi:10.1093/schbul/sbw020)
Supplement: Supplementary Data [file supp_42_6_1395__index.html]

Diagnostic Stability of ICD/DSM First Episode Psychosis Diagnoses: Meta-analysis — Diagnostic Stability of ICD/DSM First Episode Psychosis Diagnoses: Meta-analysis — Diagnostic Stability of ICD/DSM First Episode Psychosis Diagnoses: Meta-analysis — Supplementary Data 

# Diagnostic Stability of ICD/DSM First Episode Psychosis Diagnoses: Meta-analysis

## Supplementary Data

Data files

- Supplementary Data - Supplementary Data
