# Supplementary material for: Diagnostic Stability of ICD/DSM First Episode Psychosis Diagnoses: Meta-analysis
Source: Schizophr Bull. 2016 Mar 15;42(6):1395–406. doi: 10.1093/schbul/sbw020 (PMC5049518; doi:10.1093/schbul/sbw020)
Supplement: Supplementary Data [file supp_sbw020_Diagnostic_stability_supplementary_R2_clean.doc]

**Supplement**

**Diagnostic Stability of ICD/DSM First Episode Psychosis Diagnoses: Meta-analysis**

**eTable 1** Moose checklist

| **Criteria** | | **Brief description of how the criteria were handled in the meta-analysis** |
| --- | --- | --- |
| **Reporting of background should include** | |  |
| √ | Problem definition | Validity of current first episode psychosis diagnoses. We will address this at meta-analytical level. |
| √ | Hypothesis statement | Our first aim is to test the magnitude and consistency of prospective diagnostic stability of different types of first episode psychosis diagnoses, while at the same time addressing an initial first episode psychosis diagnoses changes over time (prospective diagnostic instability). We have also addressed the potential modulating effect of several confounders. |
| √ | Description of study outcomes | Baseline sample size and the number of patients with specific psychotic diagnoses at follow-up time. |
| √ | Type of exposure or intervention used | Studies included were original articles investigating patients with a first episode of psychosis, reporting the diagnosis at follow-up. Naturalistic studies, no experimental intervention used. |
| √ | Type of study designs used | Longitudinal studies. |
| √ | Study population | Subject with a first episode of psychosis defined according to international and established criteria. |
| **Reporting of search strategy should include** | |  |
| √ | Qualifications of searchers | The credentials of the two investigators MC and ZB are indicated in the author list and in the acknowledgements. |
| √ | Search strategy, including time period included in the synthesis and keywords | The search was extended until 30 June 2015, and included abstracts in English language only. The electronic research adopted several combinations of the following keywords: “First episode psychosis”, “Diagnostic accuracy”, “Sensitivity”, “Specificity”, “Psychosis prediction”, “Psychosis onset”, “Diagnostic Stability”, “Prediction”, “DSM-IV”, “ICD-10”, “Follow-up”. A second step involved the use of Scopus® and a manual search of the reference lists of the retrieved articles. |
| √ | Databases and registries searched | Web of Science^SM^, MEDLINE® and Scopus®. |
| √ | Search software used, name and version, including special features | Web of Knowledge^SM^ and Scopus®. |
| √ | Use of hand searching | We hand-searched bibliographies of retrieved papers for additional references. |
| √ | List of citations located and those excluded, including justifications | Details of the literature search process are outlined in the supplementary materials. |
| √ | Method of addressing articles published in languages other than English | The search included abstract in English language only. |
| √ | Method of handling abstracts and unpublished studies | Abstracts and unpublished studies were excluded. |
| √ | Description of any contact with authors | We contacted all the corresponding authors to provide additional data when needed. |
| **Reporting of methods should include** | |  |
| √ | Description of relevance or appropriateness of studies assembled for assessing the hypothesis to be tested | Detailed inclusion and exclusion criteria were described in the methods section. |
| √ | Rationale for the selection and coding of data | Data extracted from each of the studies were relevant to the population characteristics, study design, exposure, outcome, and possible effect of confounders. |
| √ | Assessment of confounding | Sub-groups analyses and meta-regressions were used to examine the influence of mean age, proportion of females, exposure to antipsychotics, diagnostic criteria used to assess the psychotic relapse, quality assessment, publication year, duration of untreated psychosis, co-morbid substance misuse, GAF at baseline. |
| √ | Assessment of study quality, including blinding of quality assessors; stratification or regression on possible predictors of study results | We adapted the Newcastle-Ottawa Scale for the evaluation of non-randomized studies. This tool has been adopted in recent meta-analyses. |
| √ | Assessment of heterogeneity | Heterogeneity was assessed with the I^2^ index. |
| √ | Description of statistical methods in sufficient detail to be replicated | Random effect meta-analysis conducted with the “metaprop”,”metaninf”, “metafunnel”, “metabias” packages of STATA 13.1. Description of methods of meta-analyses, sensitivity analyses, meta-regression and assessment of publication bias were fully detailed in the methods. |
| √ | Provision of appropriate tables and graphics | We included the PRISMA flow-chart and several tables to describe the literature search and its results. Several graphs were used to describe the main findings of the analyses and findings. |
| **Reporting of results should include** | |  |
| √ | Graph summarizing individual study estimates and overall estimate | We have appended them in the main text. Additional graphs were presented as supplementary material to fully describe the results. |
| √ | Table giving descriptive information for each study included | Table 1 and eTable 2 |
| √ | Results of sensitivity testing | Sensitivity analyses (i.e. exclusion of 1 study at a time) after outliers identification were reported in the main text and supplementary results when appropriate. |
| √ | Indication of statistical uncertainty of findings | We did report mean estimates for the main outcome and 95% CI. |
| **Reporting of discussion should include** | |  |
| √ | Quantitative assessment of bias | Descriptions of quantitative assessment of bias are detailed in the methods; results are described in the main text and supplementary materials. |
| √ | Justification for exclusion | Exclusion criteria were: (a) abstracts, pilot datasets, and paper in languages other than English; (b) articles that were not employing the internationally ICD/DSM validated diagnoses for psychosis; (c) articles with overlapping datasets; (d) articles which did not provide enough meta-analytical data; (e) articles reporting on organic psychoses. |
| √ | Assessment of quality of included studies | We entered the results of the Newcastle-Ottawa Quality Assessment Scale in meta-regression analyses. |
| **Reporting of conclusions should include** | |  |
| √ | Consideration of alternative explanations for observed results | We discussed alternative explanations for our findings, specifically considering potential methodological shortcomings. |
| √ | Generalization of the conclusions | We have clearly addressed the generalization of the conclusions in the discussion section |
| √ | Guidelines for future research | We have suggested possible streams of future diagnostic development and research in the discussion |
| √ | Disclosure of funding source | No separate funding was necessary for the undertaking of this systematic review. |

**eMethods**

**List of included ICD-10/DSM-IV diagnostic categories**

The clinical descriptions and diagnostic guidelines provided in the international diagnostic manuals – ICD-10 and DSM-IV – guide the differential diagnosis of patients presenting with first-episode psychosis. Here follows the nomenclature and relative codes assigned to first-episode psychoses in the two diagnostic manuals.

| **Name in the manuscript** | **Acronym in the manuscript** | **ICD-10 codes** | **ICD-10 diagnoses** | **DSM-IV codes** | **DSM-IV diagnoses** |
| --- | --- | --- | --- | --- | --- |
| Schizophrenia | SZ |  |  |  |  |
|  |  | F20.0 | Paranoid schizophrenia | 295.10 | Schizophrenia, Disorganized Type |
|  |  | F20.1 | Hebephrenic schizophrenia | 295.20 | Schizophrenia, Catatonic Type |
|  |  | F20.2 | Catatonic schizophrenia | 295.30 | Schizophrenia, Paranoid Type |
|  |  | F20.3 | Undifferentiated schizophrenia | 295.90 | Schizophrenia, Undifferentiated Type |
|  |  | F20.6 | Simple schizophrenia |  |  |
|  |  | F20.8 | Other schizophrenia |  |  |
|  |  | F20.9 | Schizophrenia, unspecified |  |  |
| Schizoaffective disorder | SA | F25 | Schizoaffective disorders | 295.70 | Schizoaffective disorder |
| Schizophreniform disorder | SF | - | - | 295.40 | Schizophreniform Disorder |
| Affective spectrum psychoses | ASP |  |  |  |  |
| *Mania with psychosis* | M | F30.2 | Mania with psychotic symptoms | 296.04 | Bipolar I disorder, single manic episode, severe with psychotic features |
| *Bipolar disorder with psychosis* | BD |  |  |  |  |
|  |  | F31.2 | Bipolar affective disorder, current episode manic with psychotic symptoms | 296.44 | Bipolar I disorder, most recent episode manic, severe with psychotic features |
|  |  | F31.5 | Bipolar affective disorder, current episode severe depression with psychotic symptoms | 296.54 | Bipolar I disorder, most recent episode depressed, severe with psychotic features |
| *Depression with psychosis* | D |  |  |  |  |
|  |  | F32.3 | Severe depressive episode with psychotic symptoms | 296.24 | Major depressive disorder, single episode, severe with psychotic features |
|  |  | F33.3 | Recurrent depressive disorder, current episode severe with psychotic symptoms | 296.34 | Major depressive disorder, recurrent, severe with psychotic features |
| Delusional disorder | DD | F22 | Persistent delusional disorders | 297.1 | Delusional disorder |
| Substance-induced psychotic disorder | SI |  |  |  |  |
|  |  | F1x.5 | Mental and behavioural disorders due to psychoactive substance use. Psychotic disorder | 291.3 | Alcohol-Induced Induced Psychotic Disorder, With Hallucinations |
|  |  |  |  | 291.5 | Alcohol-Induced Psychotic Disorder, With Delusions |
|  |  |  |  | 292.11 | Amphetamine-Induced Psychotic Disorder, With Delusions |
|  |  |  |  |  | Cannabis-Induced Psychotic Disorder, With Delusions |
|  |  |  |  |  | Cocaine-Induced Psychotic Disorder, With Delusions |
|  |  |  |  |  | Hallucinogen-Induced Psychotic Disorder, With Delusions |
|  |  |  |  |  | Inhalant-Induced Psychotic Disorder, With Delusions |
|  |  |  |  |  | Opioid-Induced Psychotic Disorder, With Delusions |
|  |  |  |  |  | Other (or Unknown) Substance Induced Psychotic Disorder, With Delusions |
|  |  |  |  |  | Phencyclidine-Induced Psychotic Disorder, With Delusions |
|  |  |  |  |  | Sedative, Hypnotic, or Anxiolytic-Induced Psychotic Disorder, With Delusions |
|  |  |  |  | 292.12 | Amphetamine-Induced Psychotic Disorder, With Hallucinations |
|  |  |  |  |  | Cannabis-Induced Psychotic Disorder, With Hallucinations |
|  |  |  |  |  | Cocaine-Induced Psychotic Disorder, With Hallucinations |
|  |  |  |  |  | Hallucinogen-Induced Psychotic Disorder, With Hallucinations |
|  |  |  |  |  | Inhalant-Induced Psychotic Disorder, With Hallucinations |
|  |  |  |  |  | Opioid-Induced Psychotic Disorder, With Hallucinations |
|  |  |  |  |  | Other (or Unknown) Substance-Induced Psychotic Disorder, With Hallucinations |
|  |  |  |  |  | Phencyclidine-Induced Psychotic Disorder, With Hallucinations |
|  |  |  |  |  | Sedative, Hypnotic, or Anxiolytic-Induced Psychotic Disorder, With Hallucinations |
| Psychosis not otherwise specified | PNOS |  |  |  |  |
|  |  | F28 | Other nonorganic psychotic disorders | 298.9 | Psychosis not otherwise specified |
|  |  | F29 | Unspecified nonorganic psychosis |  |  |
| Acute and transient psychotic disorder/brief psychotic disorder | ATPD/BPD | F23 | Acute and transient psychotic disorders | 298.8 | Brief Psychotic Disorder |

**eTable 2**. List of excluded studies

| Study name and year of publication | Reason for exclusion |
| --- | --- |
| 1. Rzewuska, et al. 1982^1^ | No internationally validated diagnosis |
| 2. Hwu, et al. 1988^2^ | DSM-III, ICD-9 |
| 3. Maj, et al. 1990^3^ | DSM-III |
| 4. Lenz, et al. 1991^4^ | DSM-III, ICD-9 |
| 5. Marneros, et al. 1991^5^ | DSM-III |
| 6. Fennig, et al. 1994^6^ | DSM-III-R |
| 7. Coryell, et al. 1995^7^ | Incomplete follow-up of baseline samples |
| 8. Cassachia, et al. 1996^8^ | DSM-III-R |
| 9. Maziade, et al. 1996^9^ | DSM-III |
| 10. Daradkeh, et al. 1997^10^ | No First Episode Psychosis |
| 11. Mason, et al. 1997^11^ | Incomplete follow-up of baseline samples |
| 12. Amin, et al. 1999^12^ | Incomplete follow-up of baseline samples |
| 13. Gold, et al. 1999^13^ | Incomplete follow-up of baseline samples |
| 14. McClellan, et al. 1999^14^ | Incomplete follow-up of baseline samples |
| 15. Robinson, et al. 1999^15^ | Incomplete follow-up of baseline samples |
| 16. Hollis. 2000^16^ | DSM-III-R |
| 17. Otote, et al. 2000^17^ | Incomplete follow-up of baseline samples |
| 18. Das, et al. 2001^18^ | Incomplete follow-up of baseline samples |
| 19. Huguelet ,et al. 2001^19^ | DSM-III-R |
| 20. Selten, et al. 2001^20^ | Incomplete follow-up of baseline samples |
| 21. Verdoux, et al. 2001^21^ | Incomplete follow-up of baseline samples |
| 22. Herberner, et al. 2002^22^ | No First Episode Psychosis |
| 23. Kalla, et al. 2002^23^ | Incomplete follow-up of baseline samples |
| 24. Drake, et al. 2003^24^ | Incomplete follow-up of baseline samples |
| 25. Marneros, et al. 2003 (a) ^25^ | Overlapping sample with Pillmann, et al. 2012 |
| 26. Marneros, et al. 2003 (b) ^26^ | Overlapping sample with Pillmann, et al. 2012 |
| 27. Harrigan, et al. 2003^27^ | DSM-III-R |
| 28. Pillmann, et al. 2003^28^ | Overlapping sample with Pillmann, et al. 2012 |
| 29. Stirling, et al. 2003^29^ | Incomplete follow-up of baseline samples |
| 30. Addington, et al. 2004^30^ | Incomplete follow-up of baseline samples |
| 31. Boks, et al. 2004^31^ | No Follow-up of baseline sample |
| 32. Singh, et al. 2004^32^ | Incomplete follow-up of baseline samples |
| 33. Veen, et al. 2004^33^ | Incomplete follow-up of baseline samples |
| 34. Baldwin, et al. 2005^34^ | Overlapping sample with Kingston et al. 2013 |
| 35. Chen, et al. 2005^35^ | Incomplete follow-up of baseline samples |
| 36. Emsley, et al. 2005^36^ | Incomplete follow-up of baseline samples |
| 37. Geller, et al. 2005^37^ | Incomplete follow-up of baseline samples |
| 38. Hlastala, et al. 2005^38^ | Incomplete follow-up of baseline samples |
| 39. Hoff, et al. 2005^39^ | Incomplete follow-up of baseline samples |
| 40. Lambert, et al. 2005^40^ | Incomplete follow-up of baseline samples |
| 41. Marneros, et al. 2005^41^ | No follow-up of baseline sample |
| 42. Negash, et al. 2005^42^ | Incomplete follow-up of baseline samples |
| 43. Kessing, et al. 2005^43^ | Incomplete follow-up of baseline samples |
| 44. Alaghband-Rad, et al. 2006^44^ | Incomplete follow-up of baseline samples |
| 45. Chaves, et al. 2006^45^ | Incomplete follow-up of baseline samples |
| 46. Malla, et al. 2006^46^ | Incomplete follow-up of baseline samples |
| 47. Ponizovsky, et al. 2006^47^ | Incomplete follow-up of baseline samples |
| 48. Velakoulis, et al. 2006^48^ | Incomplete follow-up of baseline samples |
| 49. Woo, et al. 2006^49^ | Incomplete follow-up of baseline samples |
| 50. Caton, et al. 2007^50^ | Incomplete follow-up of baseline samples |
| 51. Shaltout, et al. 2007^51^ | Incomplete follow-up of baseline samples |
| 52. Castagnini, et al. 2008^52^ | Overlapping sample with Castagnini et al. 2014 |
| 53. Correll, et al. 2008^53^ | Incomplete follow-up of baseline samples |
| 54. Petho, et al. 2008^54^ | No internationally validated diagnosis |
| 55. Rodriguez-Sanchez, et al. 2008^55^ | Incomplete follow-up of baseline samples |
| 56. van Rossum, et al. 2008^56^ | Incomplete follow-up of baseline samples |
| 57. Bogren, et al. 2009^57^ | Incomplete follow-up of baseline samples |
| 58. Crumlish, et al. 2009^58^ | Incomplete follow-up of baseline samples |
| 59. Eberhard, et al. 2009^59^ | Incomplete follow-up of baseline samples |
| 60. Lau, et al. 2009^60^ | No follow-up of baseline sample |
| 61. Brenner, et al. 2010^61^ | Incomplete follow-up of baseline samples |
| 62. Leeson, et al. 2010^62^ | Incomplete follow-up of baseline samples |
| 63. Amminger, et al. 2011^63^ | Incomplete follow-up of baseline samples |
| 64. Korver-Nieberg, et al. 2011^64^ | No follow-up of baseline sample |
| 65. Yung & Nelson. 2011^65^ | No First Episode Psychosis |
| 66. Zandi, et al. 2011^66^ | Incomplete follow-up of baseline samples |
| 67. Bromet, et al. 2011^67^ | Incomplete follow-up of baseline samples |
| 68. Atwoli, et al. 2012^68^ | Incomplete follow-up of baseline samples |
| 69. Caseiro, et al. 2012^69^ | Incomplete follow-up of baseline samples |
| 70. Green, et al. 2012^70^ | Incomplete follow-up of baseline samples |
| 71. Tohen, et al. 2012^71^ | Overlapping sample with Salvatore et al. 2009 |
| 72. Urben, et al. 2012^72^ | Incomplete follow-up of baseline samples |
| 73. Barder, et al. 2013^73^ | Incomplete follow-up of baseline samples |
| 74. Björkenstam, et al. 2013^74^ | Incomplete follow-up of baseline samples |
| 75. Castagnini, et al. 2013^75^ | No follow-up of baseline sample |
| 76. Castagnini, et al. 2013 (a)^76^ | No follow-up of baseline sample |
| 77. Asselmann, et al. 2014^77^ | No follow-up of baseline sample |
| 78. Daros, et al. 2014^78^ | No follow-up of baseline sample |
| 79. Ellersgaard, et al. 2014^79^ | Incomplete follow-up of baseline samples |
| 80. Lange, et al. 2014^80^ | Incomplete follow-up of baseline samples |
| 81. Mehta, et al. 2014^81^ | Incomplete follow-up of baseline samples |
| 82. Pandurangi, 2014^82^ | No follow-up of baseline sample |
| 83. Remberk, et al. 2014^83^ | Incomplete follow-up of baseline samples |
| 84. Rusaka, et al. 2014 (a) ^84^ | Incomplete follow-up of baseline samples |
| 85. Rusaka, et al. 2014 (b) ^85^ | Incomplete follow-up of baseline samples |
| 86. Russo, et al. 2014^86^ | Incomplete follow-up of baseline samples |
| 87. Serpa, et al. 2014^87^ | Incomplete follow-up of baseline samples |
| 88. Angst, et al. 2015^88^ | Incomplete follow-up of baseline samples |
| 89. Rajkumar, et al. 2015^89^ | No First Episode Psychosis |
| 90. Castagnini, et al. 2015^90^ | Overlapping sample with Jørgensen, et al. 1995 |

eTable 3. Duration of untreated psychosis and definition of first episode psychosis across the included studies

| Study Name and Year of publication | Duration of untreated psychosis | Definition of first episode psychosis |
| --- | --- | --- |
| 1. Okasha, et al. 1993^91^ | - 30% of cases <48 hours  - 38% >48 hours but <5 days  - 32% >5 days but < 3 months | Patients first-admitted to the psychiatric department with at least one of: hallucinations, delusions, confusion or disorientation, stupor or mutism, severe psychomotor excitement or marked hypomania or marked depression  (ICD-10 diagnostic criteria) |
| 2. Jørgensen, et al. 1995^92^ | NA | All first-admitted patients with delusional beliefs present at the time of admission or immediately prior to admission  (ICD-10 diagnostic criteria) |
| 3. Jørgensen, et al. 1997^93^ | - less than 2 weeks | Patients first-admitted to the psychiatric department, with the diagnosis of first episode ATPD  (ICD-10 diagnostic criteria) |
| 4. Das, et al. 1999^94^ | - less than 2 weeks | Consecutive patients first-admitted to the psychiatric department with the diagnosis of first episode ATPD  (ICD-10 diagnostic criteria) |
| 5. Schwartz, et al. 2000 (a)^95^ | NA | First admission patients with psychosis  (DSM-IV diagnostic criteria) |
| 6. Sajith, et al. 2002^96^ | - 66.7% of cases <48 hours  - 33.3% >48 hours but <2 weeks | Consecutive patients first-admitted to the psychiatric department with the diagnosis of first episode APPD  (ICD-10 diagnostic criteria) |
| 7. Jäger, et al. 2003^97^ | - less than 2 weeks | First-hospitalized patients with the diagnosis of ATPD  (ICD-10 diagnostic criteria) |
| 8. Jarbin, et al. 2003^98^ | - 46.3% of cases >12 weeks  - 53.7% <12 weeks | All first-admitted patients with psychosis retrospectively assessed  (DSM-IV diagnostic criteria) |
| 9. Amini, et al. 2005^99^ | -less than 3 months | First admission patients with the diagnosis of first episode psychosis  (ICD-10 or DSM-IV diagnostic criteria) |
| 10. Correll, et al. 2005^100^ | NA | First-ever presentation to any psychiatric service with the diagnosis of first episode psychosis  (DSM-IV diagnostic criteria) |
| 11. Rufino, et al. 2005^101^ | NA | Patients first-admitted to the emergency psychiatric unit with the presence of at least one of four symptom groups (delusion, hallucination, disorganized thoughts and disorganized or catatonic behaviour) for up to 6 months (BPRS, YMRS, HRSD, GAF) |
| 12. Schimmelmann, et al. 2005^102^ | - 44.6% of cases median 387 days  - 23% median 50 days  - 32.4% median 74 days | Patients first-ever admitted to EPPIC facility with a psychotic episode  (DSM-IV diagnostic criteria) |
| 13. Suda, et al. 2005^103^ | - less than 2 weeks | Patients first-ever admitted to hospital with the diagnosis of ATPD  (ICD-10 research diagnostic criteria) |
| 14. Whitty, et al. 2005^104^ | NA | First-ever presentation to any psychiatric service with the diagnosis of first episode psychosis  (DSM-IV diagnostic criteria) |
| 15. Abe, et al. 2006^105^ | - less than 2 weeks | Patients first-admitted to the psychiatric department with the diagnosis of first episode APPD  (ICD-10 diagnostic criteria) |
| 16. Addington, et al. 2006^106^ | NA | Patients first-admitted to EPP with the diagnosis of first episode psychosis (DSM-IV diagnostic criteria) |
| 17. Thangadurai, et al. 2006^107^ | - less than 2 weeks | Patients first-ever admitted to hospital with the diagnosis of ATPD  (ICD-10 diagnostic criteria) |
| 18. Boks, et al. 2006^108^ | NA | Patients first-ever admitted to the hospital with the diagnosis of first episode psychosis  (DSM-IV diagnostic criteria) |
| 19. Rahm, et al. 2007^109^ | NA | Patients first-admitted to Parachute Project with the diagnosis of first episode psychosis (DSM-IV diagnostic criteria) |
| 20. Subramaniam, et al. 2007^110^ | 22.4 months (mean) | Patients first-admitted to EPIP with the diagnosis of first episode psychosis  (DSM-IV diagnostic criteria) |
| 21. Fraguas, et al. 2008^111^ | 19.2 weeks (mean) | Patients first-admitted to the psychiatric unit with the presence of at least one of four symptom groups: delusion, hallucination, disorganized behaviour, or disorganized speech  (DSM-IV diagnostic criteria) |
| 22. Haahr, et al. 2008^112^ | 47.4 weeks (mean) | Patients first-admitted to the specialist psychiatric healthcare services of four Scandinavian health care sectors with the diagnosis of first episode psychosis  (DSM-IV diagnostic criteria) |
| 23. Chang, et al. 2009^113^ | 25.1 weeks (mean) | Patients first-admitted to EASY Project with the diagnosis of first episode psychosis  (ICD-10 diagnostic criteria) |
| 24. Crebbin, et al. 2009^114^ | NA | Patients with the diagnosis of psychotic disorder due to psychoactive substance use at their first presentation to secondary care (PACE) teams  (ICD-10 diagnostic criteria) |
| 25. Pedrós, et al. 2009^115^ | NA | Patients with the diagnosis of psychosis first-ever admitted to hospital  (DSM-IV diagnostic criteria) |
| 26. Salem, et al. 2009^116^ | - 49% of cases < 10 days  - 23% between 10 and 30 days  - 28% > 30 days | Patients first-ever admitted to the in-patient unit for ATPD and retrospectively assessed  (ICD-10 diagnostic criteria) |
| 27. Salvatore, et al. 2009^117^ | - less than 6 months | Patients first-ever admitted to hospital with the diagnosis of first episode psychosis  (DSM-IV TR diagnostic criteria) |
| 28. Aadamsoo, et al. 2011^118^ | - less than 2 weeks | Patients first-ever admitted to hospital with the diagnosis of ATPD  (ICD-10 diagnostic criteria) |
| 29. Barak, et al. 2011^119^ | - less than 4 weeks | Patients first-ever admitted to hospital with the diagnosis of first episode psychosis  (ICD-10 diagnostic criteria) |
| 30. Castro-Fornieles, et al. 2011^120^ | - less than 6 months | Patients first-ever admitted to CAFEPS with the diagnosis of first episode psychosis  (DSM-IV diagnostic criteria) |
| 31. Kim, et al. 2011^121^ | 516 days | First-ever admitted patients with the presence of at least one of four symptom groups: delusion, hallucination, disorganized or catatonic behaviour, or disorganized speech  (DSM-IV diagnostic criteria) |
| 32. Möller, et al. 2011 ^122^ | NA | First-admitted patients with the diagnosis of psychosis  (ICD-10 or DSM-IV diagnostic criteria) |
| 33. Salvatore, et al. 2011^123^ | - less than 6 months | Patients first-ever admitted to hospital with the diagnosis of first episode psychosis  (ICD-10 diagnostic criteria) |
| 34. Narayanaswamy, et al. 2012^124^ | 18.2 days (mean) | Patients first-ever admitted to hospital with the diagnosis of ATPD  (ICD-10 diagnostic criteria) |
| 35. Pillmann, et al. 2012^125^ | - less than 2 weeks | Patients first-ever admitted to hospital with the diagnosis of ATPD  (ICD-10 research diagnostic criteria) |
| 36. Kingston, et al. 2013 (a)^126^ | NA | Patients first-ever admitted to CAMFEPS with the diagnosis of first episode psychosis (DSM-IV diagnostic criteria) |
| 37. Pope, et al. 2013^127^ | NA | Patients first-ever admitted to PEPP-Montreal with the diagnosis of first episode psychosis  (DSM-IV TR diagnostic criteria) |
| 38. Castagnini & Foldager. 2014^128^ | - less than 2 weeks | Subjects listed for the first time in the DPCR with the diagnosis of ATPD  (ICD-10 research diagnostic criteria) |
| 39. Kapfhammer, et al. 2014^129^ | < 4 weeks | Patients first-ever admitted to psychiatric department with the diagnosis of first episode puerperal psychosis  (DSM-IV TR diagnostic criteria) |
| 40. Queirazza, et al. 2014^130^ | - less than 2 weeks | Patients first-ever admitted to hospital with the diagnosis of ATPD  (ICD-10 diagnostic criteria) |
| 41. Heslin, et al. 2015^131^ | 60 days (median) | Patients first-ever presented to specialist mental health services in ÆSOP-10 with the diagnosis of first episode psychosis  (ICD-10 diagnostic criteria and DSM-IV TR diagnostic criteria) |
| 42. Ranjan, et al. 2015^132^ | - less than 2 weeks | Patients first-ever admitted to hospital with the diagnosis of ATPD  (ICD-10 diagnostic criteria) |

APPD, Acute polymorphic psychotic disorder; ATPD, Acute and transient psychotic disorder; BPRS, Brief Psychiatric Rating Scale; CAFEPS, Child and Adolescent First-Episode Psychosis Study; CAMFEPS, Cavan-Monaghan First Episode Psychosis Study; DPCR, Danish Psychiatric Central Register; DSM-IV TR, Diagnostic and Statistical Manual of Mental Disorder, 4^th^ Edition Text Revised; EASY, Early Assessment Service for Young People with Psychosis; EPIP, Early Psychosis Intervention Program; EPP, Calgary Early Psychosis Program; EPPIC, Early Psychosis Prevention and Intervention Centre; GAF, Global Assessment of Function Scale; HRSD, Hamilton Rating Scale for Depression; ICD-10, International Statistical Classification of Diseases and Related Health Problems; NA, Data not available; PACE, Population-adjusted clinical epidemiology; PEPP, Prevention and Early Intervention Program for Psychoses; YMRS, Young Mania Rating Scale.

| **eTable 4. Meta-analytical cross tabulation of prospective stability and instability (4.5 yrs) of ICD-10/DSM-IV first episode affective psychosis diagnoses** | | | | | | | | | | | | | | | | | | | | | |
| --- | --- | --- | --- | --- | --- | --- | --- | --- | --- | --- | --- | --- | --- | --- | --- | --- | --- | --- | --- | --- | --- |
|  |  | *Follow-up diagnosis* | | | | | | | | | | | | | | | | | | | |
| *Baseline diagnosis* |  | **SZ** | | **SA** | | **SF** (DSM-IV only) | | **BD/M** | | **D** | | **DD** | | **ATPD/BPD** | | **SI** | | **PNOS** | | **Other** | |
|  | studies, subjects | mean | 95% CI | mean | 95% CI | mean | 95% CI | mean | 95% CI | mean | 95% CI | mean | 95% CI | mean | 95% CI | mean | 95% CI | mean | 95% CI | mean | 95% CI |
| **BD/M** (ICD-10/DSM-IV) | 18, 1084 | 0.02 | 0 - 0.08 | 0.05 | 0 - 0.11 | 0 | 0 - 0.07 | 0.9 | 0.84 - 0.96 | 0.01 | 0 - 0.06 | 0 | 0 - 0.06 | 0 | 0 - 0.06 | 0.01 | 0 - 0.06 | 0.01 | 0 - 0.06 | 0 | 0 - 0.06 |
| **D** (ICD-10/DSM-IV) | 18,  521 | 0.09 | 0.01 - 0.18 | 0.05 | 0 - 0.14 | 0 | 0 - 0.01 | 0.11 | 0.02 - 0.2 | 0.73 | 0.55 - 0.72 | 0 | 0 - 0.09 | 0 | 0 - 0.08 | 0.01 | 0 - 0.09 | 0.01 | 0 - 0.09 | 0 | 0 - 0.06 |
| The diagnostic consistency data are highlighted in light blue; BD/M, bipolar disorder with psychosis/ mania with psychosis ; D, depression with psychosis; SZ, schizophrenia; SA, schizoaffective disorder; SF, schizophreniform disorder; DD, delusional disorder; ATPD/BPD, acute and transient psychotic disorder/brief psychotic disorder; SI, substance-induced psychotic disorder; PNOS, psychosis not otherwise specified; Other, mental disorder other than psychosis. | | | | | | | | | | | | | | | | | | | | | |
|  |  |  |  |  |  |  |  |  |  |  |  |  |  |  |  |  |  |  |  |  |  |

| **eTable 5. Meta-analytical moderators of prospective diagnostic instability across each diagnostic category** | | | | | | | |
| --- | --- | --- | --- | --- | --- | --- | --- |
| *Moderator* | *n* | *Beta* | *Intercept* | *t* | *p* | *95%CI* | |
| ***SZ*** |  |  |  |  |  |  |  |
| Age (mean y) | 19 | 0.008 | -0.112 | 1,67 | 0.113 | -0.002 | 0.019 |
| Gender (% females) | 17 | 0.003 | -0.019 | 0.660 | 0.517 | -0.007 | 0.014 |
| Follow-up time (mean) | 24 | 0.001 | 0.052 | 1.780 | 0.089 | -0.001 | 0.001 |
| Co-morbid substance abuse(proportion of baseline patients) | 12 | -0.001 | 0.089 | -0.470 | 0.650 | -0.004 | 0.002 |
| Diagnostic criteria used to define baseline psychosis diagnosis (ICD-10 vs DSM-IV) | 24 | 0.025 | 0.094 | 0.430 | 0.669 | -0.096 | 0.147 |
| Publication year (y) | 24 | 0.011 | -22.740 | 2.280 | **0.033** | 0.001 | 0.022 |
| Newcastle Ottawa Scale (total score) | 24 | 0.046 | -0.211 | 1.850 | 0.078 | -0.001 | 0.097 |
| Setting (Inpatient vs Outpatient vs Emergency vs Mixed)(a) | 24 | 0.162 | 0.081 | 2.480 | **0.022** | 0.026 | 0.298 |
| Baseline functional level (GAF) | 10 | 0.001 | -0.003 | 0.410 | 0.695 | -0.006 | 0.009 |
| Duration of untreated psychosis (days) | 8 | - | - | - | - | - | . |
| ***SA*** |  |  |  |  |  |  |  |
| Age (mean y) | 15 | 0.004 | 0.247 | 0.270 | 0.791 | -0.028 | 0.036 |
| Gender (% females) | 14 | 0.004 | 0.122 | 0.300 | 0.767 | -0.029 | 0.039 |
| Follow-up time (mean) | 19 | 0.001 | 0.279 | 0.220 | 0.832 | -0.002 | 0.003 |
| Co-morbid substance abuse(proportion of baseline patients) | 11 | -0.008 | 0.539 | -1.790 | 0.108 | -0.018 | 0.002 |
| Diagnostic criteria used to define baseline psychosis diagnosis (ICD-10 vs DSM-IV) | 19 | -0.133 | 0.333 | -0.800 | 0.437 | -0.485 | 0.219 |
| Publication year (y) | 19 | -0.002 | 4.230 | -0.110 | 0.914 | -0.039 | 0.035 |
| Newcastle Ottawa Scale (total score) | 19 | 0.031 | 0.079 | 0.420 | 0.678 | -0.122 | 0.184 |
| Setting (Inpatient vs Outpatient vs Emergency vs Mixed)(a) | 19 | 0.324 | 0.186 | 1.750 | 0.099 | -0.068 | 0.718 |
| Baseline functional level (GAF) | 8 | - | - | - | - | - | . |
| Duration of untreated psychosis (days) | 7 | - | - | - | - | - | . |
| ***SF*** |  |  |  |  |  |  |  |
| Age (mean y) | 15 | 0.001 | 0.673 | 0.100 | 0.918 | -0.020 | 0.022 |
| Gender (% females) | 13 | 0.005 | 0.517 | 0.540 | 0.599 | -0.016 | 0.026 |
| Follow-up time (mean) | 20 | 0.001 | 0.678 | 0.760 | 0.458 | -0.001 | 0.003 |
| Co-morbid substance abuse(proportion of baseline patients) | 11 | -0.004 | 0.885 | -1.800 | 0.106 | -0.009 | 0.001 |
| Diagnostic criteria used to define baseline psychosis diagnosis (ICD-10 vs DSM-IV) | - | - | - | - | - | - | - |
| Publication year (y) | 20 | 0.032 | -63.116 | 2.280 | **0.035** | 0.003 | 0.061 |
| Newcastle Ottawa Scale (total score) | 20 | 0.003 | 0.690 | 0.060 | 0.955 | -0.117 | 0.123 |
| Setting (Inpatient vs Outpatient vs Emergency vs Mixed)(a) | 20 | 0.017 | 0.183 | 0.09 | 0.929 | -0.371 | 0.404 |
| Baseline functional level (GAF) | 8 | - | - | - | - | - | - |
| Duration of untreated psychosis (days) | 7 | - | - | - | - | - | - |
| ***ASP*** |  |  |  |  |  |  |  |
| Age (mean y) | 18 | 0.001 | 0.147 | 0.060 | 0.957 | -0.010 | 0.010 |
| Gender (% females) | 17 | -0.002 | 0.218 | -0.280 | 0.783 | -0.013 | 0.010 |
| Follow-up time (mean) | 23 | 0.001 | 0.112 | 1.760 | 0.093 | -0.001 | 0.002 |
| Co-morbid substance abuse(proportion of baseline patients) | 13 | -0.001 | 0.165 | -0.640 | 0.536 | -0.004 | 0.002 |
| Diagnostic criteria used to define baseline psychosis diagnosis (ICD-10 vs DSM-IV) | 23 | 0.004 | 0.164 | 0.060 | 0.953 | -0.126 | 0.133 |
| Publication year (y) | 23 | 0.004 | -8.275 | 0.780 | 0.442 | -0.007 | 0.015 |
| Newcastle Ottawa Scale (total score) | 23 | 0.014 | 0.067 | 0.530 | 0.604 | -0.040 | 0.068 |
| Setting (Inpatient vs Outpatient vs Emergency vs Mixed)(a) | 23 | 0.151 | 0.065 | 2.310 | **0.032** | 0.014 | 0.287 |
| Baseline functional level (GAF) | 9 | - | - | - | - | - | - |
| Duration of untreated psychosis (days) | 8 | - | - | - | - | - | - |
| ***DD*** |  |  |  |  |  |  |  |
| Age (mean y) | 16 | 0.011 | 0.096 | 1.550 | 0.144 | -0.004 | 0.025 |
| Gender (% females) | 15 | 0.006 | 0.181 | 0.410 | 0.687 | -0.026 | 0.038 |
| Follow-up time (mean) | 22 | 0.001 | 0.333 | 0.920 | 0.369 | -0.001 | 0.003 |
| Co-morbid substance abuse(proportion of baseline patients) | 9 | - | - | - | - | - | - |
| Diagnostic criteria used to define baseline psychosis diagnosis (ICD-10 vs DSM-IV) | 22 | -0.006 | 0.414 | -0.050 | 0.964 | -0.289 | 0.277 |
| Publication year (y) | 22 | 0.031 | -60.940 | 2.160 | **0.043** | 0.001 | 0.060 |
| Newcastle Ottawa Scale (total score) | 22 | 0.044 | 0.105 | 0.680 | 0.503 | -0.090 | 0.179 |
| Setting (Inpatient vs Outpatient vs Emergency vs Mixed)(a) | 22 | 0.501 | 0.307 | 3.01 | **0.007** | 0.151 | 0.849 |
| Baseline functional level (GAF) | 7 | - | - | - | - | - | - |
| Duration of untreated psychosis (days) | 6 | - | - | - | - | - | - |
| ***ATPD/BPD*** |  |  |  |  |  |  |  |
| Age (mean y) | 30 | 0.003 | 0.315 | 0.350 | 0.726 | -0.012 | 0.017 |
| Gender (% females) | 29 | -0.005 | 0.654 | -1.500 | 0.146 | -0.012 | 0.001 |
| Follow-up time (mean) | 40 | 0.001 | 0.356 | 1.380 | 0.174 | -0.001 | 0.002 |
| Co-morbid substance abuse(proportion of baseline patients) | 15 | -0.002 | 0.502 | -0.780 | 0.448 | -0.009 | 0.004 |
| Diagnostic criteria used to define baseline psychosis diagnosis (ICD-10 vs DSM-IV) | 40 | -0.131 | 0.507 | -1.650 | 0.107 | -0.291 | 0.029 |
| Publication year (y) | 40 | 0.013 | -27.690 | 3.140 | **0.003*** | 0.004 | 0.023 |
| Newcastle Ottawa Scale (total score) | 40 | 0.068 | -0.016 | 5.610 | **0.001*** | 0.043 | 0.092 |
| Setting (Inpatient vs Outpatient vs Emergency vs Mixed)(a) | 40 | 0.219 | 0.069 | 3.190 | **0.003*** | 0.079 | 0.358 |
| Baseline functional level (GAF) | 15 | -0.003 | 0.559 | -0.770 | 0.455 | -0.011 | 0.005 |
| Duration of untreated psychosis (days) | 8 | - | - | - | - | - | - |
| ***SI*** |  |  |  |  |  |  |  |
| Age (mean y) | 9 | - | - | - | - | - | - |
| Gender (% females) | 7 | - | - | - | - | - | - |
| Follow-up time (mean) | 10 | -0.001 | 0.384 | -0.420 | 0.689 | -0.005 | 0.003 |
| Co-morbid substance abuse(proportion of baseline patients) | 5 | - | - | - | - | - | - |
| Diagnostic criteria used to define baseline psychosis diagnosis (ICD-10 vs DSM-IV) | 10 | -0.087 | 0.372 | -0.530 | 0.609 | -0.461 | 0.288 |
| Publication year (y) | 10 | -0.005 | 10.241 | -0.360 | 0.732 | -0.037 | 0.027 |
| Newcastle Ottawa Scale (total score) | 10 | -0.067 | 0.829 | -0.820 | 0.435 | -0.256 | 0.122 |
| Setting (Inpatient vs Outpatient vs Emergency vs Mixed)(a) | 10 | 0.053 | 0.176 | 0.300 | 0.770 | -0.362 | 0.470 |
| Baseline functional level (GAF) | 2 | - | - | - | - | - | - |
| Duration of untreated psychosis (days) | 5 | - | - | - | - | - | - |
| ***PNOS*** |  |  |  |  |  |  |  |
| Age (mean y) | 20 | -0.001 | 0.641 | -0.040 | 0.970 | -0.005 | 0.005 |
| Gender (% females) | 18 | -0.001 | 0.727 | -0.300 | 0.770 | -0.015 | 0.011 |
| Follow-up time (mean) | 24 | 0.001 | 0.589 | 0.980 | 0.339 | -0.001 | 0.004 |
| Co-morbid substance abuse(proportion of baseline patients) | 11 | -0.006 | 0.756 | -1.450 | 0.182 | -0.014 | 0.003 |
| Diagnostic criteria used to define baseline psychosis diagnosis (ICD-10 vs DSM-IV) | 24 | 0.032 | 0.628 | 0.320 | 0.753 | -0.181 | 0.247 |
| Publication year (y) | 24 | 0.006 | -11.969 | 0.500 | 0.621 | -0.019 | 0.032 |
| Newcastle Ottawa Scale (total score) | 24 | -0.040 | 0.903 | -1.000 | 0.330 | -0.126 | 0.044 |
| Setting (Inpatient vs Outpatient vs Emergency vs Mixed)(a) | 24 | 0.136 | 0.596 | 1.010 | 0.322 | -0.142 | 0.414 |
| Baseline functional level (GAF) | 7 | - | - | - | - | - | - |
| Duration of untreated psychosis (days) | 7 | - | - | - | - | - | - |
| SZ, schizophrenia; SA, schizoaffective disorder; SF, Schizophreniform disorder; ASP, affective spectrum psychoses; DD, delusional disorder; ATPD/BPD, acute and transient psychotic disorder/brief psychotic disorder; SI, substance-induced psychotic disorder; PNOS, psychosis not otherwise specified.  (a) Inpatient vs Mixed (inpatient and/or outpatient and/or emergency);  * significant at an alpha level of 0.01 | | | | | | | |

**eFigure 1**. Publication biases. Funnel plot of diagnostic instability (n=45 studies) and metatrim analysis.

**eFigure 2a**. Sensitivity analysis of diagnostic instability in ICD-10 studies.

**eFigure 2b**. Sensitivity analysis of diagnostic instability in DSM-IV studies

| **eTable 6. Meta-analytical cross tabulation of retrospective diagnostic stability and instability (average 4.5 yrs) ICD-10/DSM-IV first episode psychosis diagnoses** | | | | | | | | | | | | | | | | | |
| --- | --- | --- | --- | --- | --- | --- | --- | --- | --- | --- | --- | --- | --- | --- | --- | --- | --- |
|  |  | *Baseline diagnosis* | | | | | | | | | | | | | | | |
|  |  | **SZ** | | **SA** | | **SF** (DSM only) | | **ASP** | | **DD** | | **ATPD/BPD** | | **SI** | | **PNOS** | |
| *Follow up diagnosis* | studies, subjects | mean | 95% CI | mean | 95% CI | mean | 95% CI | mean | 95% CI | mean | 95% CI | mean | 95% CI | mean | 95% CI | mean | 95% CI |
| **SZ** (ICD/DSM) | 44, 4526 | 0.5 | 0.44 - 0.57 | 0.01 | 0 - 0.08 | 0.16 | 0.10 - 0.28 | 0.02 | 0 - 0.09 | 0.01 | 0 - 0.09 | 0.24 | 0.17 - 0.31 | 0.01 | 0 - 0.08 | 0.05 | 0 - 0.13 |
| **SA** (ICD/DSM**)** | 28, 578 | 0.13 | 0.04 - 0.22 | 0.39 | 0.30 - 0.48 | 0.07 | 0 - 0.19 | 0.16 | 0.07 - 0.25 | 0.01 | 0 - 0.11 | 0.16 | 0.08 - 0.26 | 0.01 | 0 - 0.10 | 0.07 | 0 - 0.17 |
| **SF** (DSM only) | 19, 193 | 0.04 | 0 - 0.18 | 0.01 | 0 - 0.14 | 0.88 | 0,75 - 1 | 0.01 | 0 - 0.15 | 0 | 0 - 0.14 | 0.02 | 0 - 0.16 | 0.01 | 0 - 0.15 | 0.03 | 0 - 0.17 |
| **ASP** (ICD/DSM) | 37, 2809 | 0.04 | 0 - 0.15 | 0.01 | 0 - 0.09 | 0.03 | 0 - 0.10 | 0.64 | 0.57 - 0.62 | 0.01 | 0 - 0.08 | 0.24 | 0.16 -0.31 | 0 | 0 - 0.08 | 0.03 | 0 - 0.10 |
| **DD** (ICD/DSM) | 27, 295 | 0 | 0 - 0.15 | 0 | 0 - 0.14 | 0.01 | 0 - 0.22 | 0.04 | 0 - 0.19 | 0.64 | 0.50 - 0.79 | 0.23 | 0.09 - 0.37 | 0.02 | 0 - 0.16 | 0.06 | 0 - 0.19 |
| **ATPD/BPD** (ICD/DSM**)** | 39, 4838 | 0 | 0 - 0.03 | 0 | 0 - 0.03 | 0.01 | 0 - 0.19 | 0 | 0 - 0.03 | 0 | 0 - 0.03 | 0.99 | 0.96 -1 | 0 | 0 - 0.03 | 0 | 0 - 0.03 |
| **SI** (ICD/DSM) | 12, 265 | 0.05 | 0 - 0.21 | 0.01 | 0 - 0.19 | 0.06 | 0 - 0.29 | 0.06 | 0 - 0.23 | 0 | 0 - 0.18 | 0.26 | 0.98 - 0.43 | 0.53 | 0.36 - 0.70 | 0.03 | 0 - 0.20 |
| **PNOS** (ICD/DSM) | 27, 345 | 0.06 | 0 - 0.19 | 0.02 | 0 - 0.14 | 0.03 | 0 - 0.19 | 0.06 | 0 - 0.19 | 0.01 | 0 - 0.13 | 0.24 | 0.12 - 0.37 | 0.01 | 0 - 0.14 | 0.57 | 0.44 - 0.69 |
| **Other** (ICD/DSM) | 14, 635 | 0.02 | 0 - 0.19 | 0.01 | 0 - 0.19 | 0 | 0 - 0.21 | 0.21 | 0.03 - 0.28 | 0 | 0 - 0.18 | 0.57 | 0.40 - 0.75 | 0.01 | 0 - 0.18 | 0.18 | 0.01 - 0.35 |
| The diagnostic stability data are highlighted in light blue; SZ, schizophrenia; SA, schizoaffective disorder; SF, schizophreniform disorder; ASP, affective spectrum psychoses; DD, delusional disorder; ATPD/BPD, acute and transient psychotic episode/brief psychotic disorder; SI, substance-induced psychotic disorder; PNOS, psychosis not otherwise specified; Other, mental disorder other than psychosis; | | | | | | | | | | | | | | | | | |
|

| **eTable 7. Meta-analytical cross tabulation of retrospective diagnostic stability and instability (average 4.5 yrs) of ICD-10/DSM-IV first episode schizophrenia spectrum and affective spectrum psychoses** | | | | | | | | | | | | | | | |
| --- | --- | --- | --- | --- | --- | --- | --- | --- | --- | --- | --- | --- | --- | --- | --- |
|  |  | *Follow-up diagnosis* | | | | | | | | | | | | | |
| *Baseline diagnosis* |  | **Schizophrenia spectrum psychoses** | | **Affective spectrum**  **psychoses** | | **DD** | | **ATPD/BPD** | | **SI** | | **PNOS** | | **Other** | |
|  | studies, subjects | mean | 95% CI | mean | 95% CI | mean | 95% CI | mean | 95% CI | mean | 95% CI | mean | 95% CI | mean | 95% CI |
| **Schizophrenia spectrum psychoses** (ICD10/DSM-IV) | 44, 5297 | 0.6 | 0.53 - 0.68 | 0.04 | 0 - 0.11 | 0.03 | 0 - 0.10 | 0.23 | 0.17 - 0.31 | 0.04 | 0 - 0.11 | 0.06 | 0 - 0.14 | 0 | 0 |
| **Affective spectrum psychoses** (ICD10/DSM-IV) | 37, 2809 | 0.08 | 0 - 0.16 | 0.64 | 0.56 - 0.72 | 0.01 | 0 - 0.09 | 0.24 | 0.15 - 0.32 | 0.01 | 0 - 0.01 | 0.02 | 0 - 0.11 | 0 | 0 - 0.08 |
| The diagnostic stability data are highlighted in light blue; Schizophrenia spectrum psychoses: schizoaffective disorders, schizophreniform disorder, schizophrenia; DD, delusional disorder; ATPD/BPD, acute and transient psychotic disorder/brief psychotic disorder; SI, substance-inducedpsychotic disorder; PNOS, psychosis not otherwise specified; Other, mental disorder other than psychosis. | | | | | | | | | | | | | | | |
|  |  |  |  |  |  |  |  |  |  |  |  |  |  |  |  |

**REFERENCES TO SUPPLEMENTARY MATERIAL**

**1.** Rzewuska M, Angst J. Aspects of the course of bipolar manic-depressive, schizo-affective, and paranoid schizophrenic psychoses. *Archiv fur Psychiatrie und Nervenkrankheiten* 1982;231(6):487-501.

**2.** Hwu HG, Chen CC, Strauss JS, Tan KL, Tsuang MT, Tseng WS. A comparative study on schizophrenia diagnosed by ICD-9 and DSM-III: course, family history and stability of diagnosis. *Acta psychiatrica Scandinavica* Jan 1988;77(1):87-97.

**3.** Maj M, Pirozzi R, Di Caprio EL. Major depression with mood-congruent psychotic features: a distinct diagnostic entity or a more severe subtype of depression? *Acta psychiatrica Scandinavica* Dec 1990;82(6):439-444.

**4.** Lenz G, Simhandl C, Thau K, Berner P, Gabriel E. Temporal stability of diagnostic criteria for functional psychoses. Results from the Vienna follow-up study. *Psychopathology* 1991;24(5):328-335.

**5.** Marneros A, Deister A, Rohde A. Stability of diagnoses in affective, schizoaffective and schizophrenic disorders. Cross-sectional versus longitudinal diagnosis. *European archives of psychiatry and clinical neuroscience* 1991;241(3):187-192.

**6.** Fennig S, Kovasznay B, Rich C, et al. Six-month stability of psychiatric diagnoses in first-admission patients with psychosis. *The American journal of psychiatry* Aug 1994;151(8):1200-1208.

**7.** Coryell W, Endicott J, Maser JD, Keller MB, Leon AC, Akiskal HS. Long-term stability of polarity distinctions in the affective disorders. *The American journal of psychiatry* Mar 1995;152(3):385-390.

**8.** Casacchia M, de Cataldo S, Roncone R, Marcelli G. Schizophreniform disorder: a 1-year follow-up study. *Psychopathology* 1996;29(2):104-108.

**9.** Maziade M, Bouchard S, Gingras N, et al. Long-term stability of diagnosis and symptom dimensions in a systematic sample of patients with onset of schizophrenia in childhood and early adolescence. II: Postnegative distinction and childhood predictors of adult outcome. *The British journal of psychiatry : the journal of mental science* Sep 1996;169(3):371-378.

**10.** Daradkeh T, El-Rufaie O, Younis Y, Ghubash R. The diagnostic stability of ICD-10 psychiatric diagnoses in clinical practice. *European psychiatry : the journal of the Association of European Psychiatrists* 1997;12(3):136-139.

**11.** Mason P, Harrison G, Croudace T, Glazebrook C, Medley I. The predictive validity of a diagnosis of schizophrenia. A report from the International Study of Schizophrenia (ISoS) coordinated by the World Health Organization and the Department of Psychiatry, University of Nottingham. *The British journal of psychiatry : the journal of mental science* Apr 1997;170:321-327.

**12.** Amin S, Singh SP, Brewin J, Jones PB, Medley I, Harrison G. Diagnostic stability of first-episode psychosis. Comparison of ICD-10 and DSM-III-R systems. *The British journal of psychiatry : the journal of mental science* Dec 1999;175:537-543.

**13.** Gold S, Arndt S, Nopoulos P, O'Leary DS, Andreasen NC. Longitudinal study of cognitive function in first-episode and recent-onset schizophrenia. *The American journal of psychiatry* Sep 1999;156(9):1342-1348.

**14.** McClellan J, McCurry C. Early onset psychotic disorders: diagnostic stability and clinical characteristics. *European child & adolescent psychiatry* 1999;8 Suppl 1:I13-19.

**15.** Robinson D, Woerner MG, Alvir JM, et al. Predictors of relapse following response from a first episode of schizophrenia or schizoaffective disorder. *Arch Gen Psychiatry* Mar 1999;56(3):241-247.

**16.** Hollis C. Adult outcomes of child- and adolescent-onset schizophrenia: diagnostic stability and predictive validity. *The American journal of psychiatry* Oct 2000;157(10):1652-1659.

**17.** Otote DI, Ohaeri JU. Depressive symptomatology and short-term stability at a Nigerian psychiatric care facility. *Psychopathology* Nov-Dec 2000;33(6):314-323.

**18.** Das SK, Malhotra S, Basu D, Malhotra R. Testing the stress-vulnerability hypothesis in ICD-10-diagnosed acute and transient psychotic disorders. *Acta psychiatrica Scandinavica* Jul 2001;104(1):56-58.

**19.** Huguelet P, Schneider El Gueddari N, Glauser D. Stability of DSM-III-R diagnoses: study of a case register. *Psychopathology* May-Jun 2001;34(3):118-122.

**20.** Selten JP, Veen N, Feller W, et al. Incidence of psychotic disorders in immigrant groups to The Netherlands. *The British journal of psychiatry : the journal of mental science* Apr 2001;178:367-372.

**21.** Verdoux H, Liraud F, Bergey C, Assens F, Abalan F, van Os J. Is the association between duration of untreated psychosis and outcome confounded? A two year follow-up study of first-admitted patients. *Schizophrenia research* Apr 30 2001;49(3):231-241.

**22.** Herbener ES, Harrow M. The course of anhedonia during 10 years of schizophrenic illness. *Journal of abnormal psychology* May 2002;111(2):237-248.

**23.** Kalla O, Aaltonen J, Wahlstrom J, Lehtinen V, Garcia Cabeza I, Gonzalez de Chavez M. Duration of untreated psychosis and its correlates in first-episode psychosis in Finland and Spain. *Acta psychiatrica Scandinavica* Oct 2002;106(4):265-275.

**24.** Drake RJ, Dunn G, Tarrier N, Haddock G, Haley C, Lewis S. The evolution of symptoms in the early course of non-affective psychosis. *Schizophrenia research* Sep 1 2003;63(1-2):171-179.

**25.** Marneros A, Pillmann F, Haring A, Balzuweit S, Bloink R. What is schizophrenic in acute and transient psychotic disorder? *Schizophr Bull* 2003;29(2):311-323.

**26.** Marneros A, Pillmann F, Haring A, Balzuweit S, Bloink R. Features of acute and transient psychotic disorders. *European archives of psychiatry and clinical neuroscience* Aug 2003;253(4):167-174.

**27.** Harrigan SM, McGorry PD, Krstev H. Does treatment delay in first-episode psychosis really matter? *Psychological medicine* Jan 2003;33(1):97-110.

**28.** Pillmann F, Haring A, Balzuweit S, Bloink R, Marneros A. Bouffee delirante and ICD-10 acute and transient psychoses: a comparative study. *The Australian and New Zealand journal of psychiatry* Jun 2003;37(3):327-333.

**29.** Stirling J, White C, Lewis S, Hopkins R, Tantam D, Huddy A, Montague L. Neurocognitive function and outcome in first-episode schizophrenia: a 10-year follow-up of an epidemiological cohort. *Schizophrenia research* Dec 15 2003;65(2-3):75-86.

**30.** Addington J, Van Mastrigt S, Addington D. Duration of untreated psychosis: impact on 2-year outcome. *Psychological medicine* Feb 2004;34(2):277-284.

**31.** Boks MP, Liddle PF, Burgerhof JG, Knegtering R, van den Bosch RJ. Neurological soft signs discriminating mood disorders from first episode schizophrenia. *Acta psychiatrica Scandinavica* Jul 2004;110(1):29-35.

**32.** Singh SP, Burns T, Amin S, Jones PB, Harrison G. Acute and transient psychotic disorders: precursors, epidemiology, course and outcome. *The British journal of psychiatry : the journal of mental science* Dec 2004;185:452-459.

**33.** Veen ND, Selten JP, Schols D, Laan W, Hoek HW, van der Tweel I, Kahn RS. Diagnostic stability in a Dutch psychosis incidence cohort. *The British journal of psychiatry : the journal of mental science* Dec 2004;185:460-464.

**34.** Baldwin P, Browne D, Scully PJ, et al. Epidemiology of first-episode psychosis: illustrating the challenges across diagnostic boundaries through the Cavan-Monaghan study at 8 years. *Schizophr Bull* Jul 2005;31(3):624-638.

**35.** Chen EY, Tam DK, Wong JW, Law CW, Chiu CP. Self-administered instrument to measure the patient's experience of recovery after first-episode psychosis: development and validation of the Psychosis Recovery Inventory. *The Australian and New Zealand journal of psychiatry* Jun 2005;39(6):493-499.

**36.** Emsley R, Turner HJ, Oosthuizen PP, Carr J. Neurological abnormalities in first-episode schizophrenia: temporal stability and clinical and outcome correlates. *Schizophrenia research* Jun 1 2005;75(1):35-44.

**37.** Geller B, Tillman R. Prepubertal and early adolescent bipolar I disorder: review of diagnostic validation by Robins and Guze criteria. *The Journal of clinical psychiatry* 2005;66 Suppl 7:21-28.

**38.** Hlastala SA, McClellan J. Phenomenology and diagnostic stability of youths with atypical psychotic symptoms. *Journal of child and adolescent psychopharmacology* Jun 2005;15(3):497-509.

**39.** Hoff AL, Svetina C, Shields G, Stewart J, DeLisi LE. Ten year longitudinal study of neuropsychological functioning subsequent to a first episode of schizophrenia. *Schizophrenia research* Oct 1 2005;78(1):27-34.

**40.** Lambert M, Conus P, Lubman DI, et al. The impact of substance use disorders on clinical outcome in 643 patients with first-episode psychosis. *Acta psychiatrica Scandinavica* Aug 2005;112(2):141-148.

**41.** Marneros A, Pillmann F, Haring A, Balzuweit S, Bloink R. Is the psychopathology of acute and transient psychotic disorder different from schizophrenic and schizoaffective disorders? *European psychiatry : the journal of the Association of European Psychiatrists* Jun 2005;20(4):315-320.

**42.** Negash A, Alem A, Kebede D, Deyessa N, Shibre T, Kullgren G. Prevalence and clinical characteristics of bipolar I disorder in Butajira, Ethiopia: a community-based study. *Journal of affective disorders* Aug 2005;87(2-3):193-201.

**43.** Kessing LV. Diagnostic stability in bipolar disorder in clinical practise as according to ICD-10. *Journal of affective disorders* Apr 2005;85(3):293-299.

**44.** Alaghband-Rad J, Boroumand M, Amini H, et al. Non-affective Acute Remitting Psychosis: a preliminary report from Iran. *Acta psychiatrica Scandinavica* Feb 2006;113(2):96-101.

**45.** Chaves AC, Addington J, Seeman M, Addington D. One-year stability of diagnosis in first-episode nonaffective psychosis: influence of sex. *Canadian journal of psychiatry Revue canadienne de psychiatrie* Oct 2006;51(11):711-714.

**46.** Malla A, Norman R, Schmitz N, Manchanda R, Bechard-Evans L, Takhar J, Haricharan R. Predictors of rate and time to remission in first-episode psychosis: a two-year outcome study. *Psychological medicine* May 2006;36(5):649-658.

**47.** Ponizovsky AM, Grinshpoon A, Pugachev I, Nahon D, Ritsner M, Abramowitz MZ. Changes in stability of first-admission psychiatric diagnoses over 14 years, based on cross-sectional data at three time points. *The Israel journal of psychiatry and related sciences* 2006;43(1):34-39.

**48.** Velakoulis D, Wood SJ, Wong MT, et al. Hippocampal and amygdala volumes according to psychosis stage and diagnosis: a magnetic resonance imaging study of chronic schizophrenia, first-episode psychosis, and ultra-high-risk individuals. *Arch Gen Psychiatry* Feb 2006;63(2):139-149.

**49.** Woo BK, Sevilla CC, Obrocea GV. Factors influencing the stability of psychiatric diagnoses in the emergency setting: review of 934 consecutively inpatient admissions. *General hospital psychiatry* Sep-Oct 2006;28(5):434-436.

**50.** Caton CL, Hasin DS, Shrout PE, Drake RE, Dominguez B, First MB, Samet S, Schanzer B. Stability of early-phase primary psychotic disorders with concurrent substance use and substance-induced psychosis. *The British journal of psychiatry : the journal of mental science* Feb 2007;190:105-111.

**51.** Shaltout T, Bener A, Al Abdullah M, Al Mujalli Z, Shaltout HT. Acute and transient psychotic disorders in a rapidly developing country, State of Qatar. *Medicina* 2007;43(7):575-579.

**52.** Castagnini A, Bertelsen A, Berrios GE. Incidence and diagnostic stability of ICD-10 acute and transient psychotic disorders. *Comprehensive psychiatry* May-Jun 2008;49(3):255-261.

**53.** Correll CU, Smith CW, Auther AM, et al. Predictors of remission, schizophrenia, and bipolar disorder in adolescents with brief psychotic disorder or psychotic disorder not otherwise specified considered at very high risk for schizophrenia. *Journal of child and adolescent psychopharmacology* Oct 2008;18(5):475-490.

**54.** Petho B, Tolna J, Tusnady G, Farkas M, Vizkeleti G, Vargha A, Czobor P. The predictive validity of the Leonhardean classification of endogenous psychoses: a 21-33-year follow-up of a prospective study ("BUDAPEST 2000"). *European archives of psychiatry and clinical neuroscience* Sep 2008;258(6):324-334.

**55.** Rodriguez-Sanchez JM, Perez-Iglesias R, Gonzalez-Blanch C, et al. 1-year follow-up study of cognitive function in first-episode non-affective psychosis. *Schizophrenia research* Sep 2008;104(1-3):165-174.

**56.** van Rossum I, Haro JM, Tenback D, Boomsma M, Goetz I, Vieta E, van Os J, Board EA. Stability and treatment outcome of distinct classes of mania. *European psychiatry : the journal of the Association of European Psychiatrists* Aug 2008;23(5):360-367.

**57.** Bogren M, Mattisson C, Isberg PE, Nettelbladt P. How common are psychotic and bipolar disorders? A 50-year follow-up of the Lundby population. *Nordic journal of psychiatry* 2009;63(4):336-346.

**58.** Crumlish N, Whitty P, Clarke M, et al. Beyond the critical period: longitudinal study of 8-year outcome in first-episode non-affective psychosis. *The British journal of psychiatry : the journal of mental science* Jan 2009;194(1):18-24.

**59.** Eberhard J, Levander S, Lindstrom E. Remission in schizophrenia: analysis in a naturalistic setting. *Comprehensive psychiatry* May-Jun 2009;50(3):200-208.

**60.** Lau PW, Cheng JG, Chow DL, Ungvari GS, Leung CM. Acute psychiatric disorders in foreign domestic workers in Hong Kong: a pilot study. *The International journal of social psychiatry* Nov 2009;55(6):569-576.

**61.** Brenner I, Krivoy A, Weizman A, Fischel T. Stability of schizoaffective disorder in correlation with duration of follow-up: retrospective analysis. *Psychopathology* 2010;43(5):285-291.

**62.** Leeson VC, Barnes TR, Harrison M, Matheson E, Harrison I, Mutsatsa SH, Ron MA, Joyce EM. The relationship between IQ, memory, executive function, and processing speed in recent-onset psychosis: 1-year stability and clinical outcome. *Schizophr Bull* Mar 2010;36(2):400-409.

**63.** Amminger GP, Henry LP, Harrigan SM, Harris MG, Alvarez-Jimenez M, Herrman H, Jackson HJ, McGorry PD. Outcome in early-onset schizophrenia revisited: findings from the Early Psychosis Prevention and Intervention Centre long-term follow-up study. *Schizophrenia research* Sep 2011;131(1-3):112-119.

**64.** Korver-Nieberg N, Quee PJ, Boos HB, Simons CJ, Group. The validity of the DSM-IV diagnostic classification system of non-affective psychoses. *The Australian and New Zealand journal of psychiatry* Dec 2011;45(12):1061-1068.

**65.** Yung AR, Nelson B. Young people at ultra high risk for psychosis: research from the PACE clinic. *Revista brasileira de psiquiatria* Oct 2011;33 Suppl 2:s143-160.

**66.** Zandi T, Havenaar JM, Laan W, Kahn RS, van den Brink W. Predictive validity of a culturally informed diagnosis of schizophrenia: a 30 month follow-up study with first episode psychosis. *Schizophrenia research* Dec 2011;133(1-3):29-35.

**67.** Bromet EJ, Kotov R, Fochtmann LJ, Carlson GA, Tanenberg-Karant M, Ruggero C, Chang SW. Diagnostic shifts during the decade following first admission for psychosis. *The American journal of psychiatry* Nov 2011;168(11):1186-1194.

**68.** Atwoli L, Ndambuki D, Owiti P, Manguro G, Omulimi N. Short-term diagnostic stability among re-admitted psychiatric in-patients in Eldoret, Kenya. *African journal of psychiatry* Mar 2012;15(2):114-118.

**69.** Caseiro O, Perez-Iglesias R, Mata I, et al. Predicting relapse after a first episode of non-affective psychosis: a three-year follow-up study. *Journal of psychiatric research* Aug 2012;46(8):1099-1105.

**70.** Green MF, Bearden CE, Cannon TD, et al. Social cognition in schizophrenia, Part 1: performance across phase of illness. *Schizophr Bull* Jun 2012;38(4):854-864.

**71.** Tohen M, Khalsa HM, Salvatore P, Vieta E, Ravichandran C, Baldessarini RJ. Two-year outcomes in first-episode psychotic depression the McLean-Harvard First-Episode Project. *Journal of affective disorders* Jan 2012;136(1-2):1-8.

**72.** Urben S, Baumann P, Barcellona S, et al. Cognitive efficacy of quetiapine in early-onset first-episode psychosis: a 12-week open label trial. *The Psychiatric quarterly* Sep 2012;83(3):311-324.

**73.** Barder HE, Sundet K, Rund BR, et al. Neurocognitive development in first episode psychosis 5 years follow-up: associations between illness severity and cognitive course. *Schizophrenia research* Sep 2013;149(1-3):63-69.

**74.** Bjorkenstam E, Bjorkenstam C, Hjern A, Reutfors J, Boden R. A five year diagnostic follow-up of 1,840 patients after a first episode non-schizophrenia and non-affective psychosis. *Schizophrenia research* Oct 2013;150(1):205-210.

**75.** Castagnini A, Foldager L. Variations in incidence and age of onset of acute and transient psychotic disorders. *Social psychiatry and psychiatric epidemiology* Dec 2013;48(12):1917-1922.

**76.** Castagnini AC, Laursen TM, Mortensen PB, Bertelsen A. Family psychiatric morbidity of acute and transient psychotic disorders and their relationship to schizophrenia and bipolar disorder. *Psychological medicine* Nov 2013;43(11):2369-2375.

**77.** Asselmann E, Wittchen HU, Lieb R, Hofler M, Beesdo-Baum K. Associations of fearful spells and panic attacks with incident anxiety, depressive, and substance use disorders: a 10-year prospective-longitudinal community study of adolescents and young adults. *Journal of psychiatric research* Aug 2014;55:8-14.

**78.** Daros AR, Ruocco AC, Reilly JL, Harris MS, Sweeney JA. Facial emotion recognition in first-episode schizophrenia and bipolar disorder with psychosis. *Schizophrenia research* Mar 2014;153(1-3):32-37.

**79.** Ellersgaard D, Mors O, Thorup A, Jorgensen P, Jeppesen P, Nordentoft M. Prospective study of the course of delusional themes in first-episode non-affective psychosis. *Early intervention in psychiatry* Nov 2014;8(4):340-347.

**80.** Lange EH, Nesvag R, Ringen PA, Hartberg CB, Haukvik UK, Andreassen OA, Melle I, Agartz I. One year follow-up of alcohol and illicit substance use in first-episode psychosis: does gender matter? *Comprehensive psychiatry* Feb 2014;55(2):274-282.

**81.** Mehta S, Tyagi A, Swami MK, Gupta S, Kumar M, Tripathi R. Onset of acute and transient psychotic disorder in India: a study of socio-demographics and factors affecting its outcomes. *East Asian archives of psychiatry : official journal of the Hong Kong College of Psychiatrists = Dong Ya jing shen ke xue zhi : Xianggang jing shen ke yi xue yuan qi kan* Jun 2014;24(2):75-80.

**82.** Pandurangi AK. Psychotic disorders in Asian Americans and DSM-5. *Asian journal of psychiatry* Feb 2014;7(1):83-85.

**83.** Remberk B, Bazynska AK, Krempa-Kowalewska A, Rybakowski F. Adolescent insanity revisited: course and outcome in early-onset schizophrenia spectrum psychoses in an 8-year follow-up study. *Comprehensive psychiatry* Jul 2014;55(5):1174-1181.

**84.** Rusaka M, Rancans E. A prospective follow-up study of first-episode acute transient psychotic disorder in Latvia. *Annals of general psychiatry* 2014;13(1):4.

**85.** Rusaka M, Rancans E. First-episode acute and transient psychotic disorder in Latvia: a 6-year follow-up study. *Nordic journal of psychiatry* Jan 2014;68(1):24-29.

**86.** Russo M, Levine SZ, Demjaha A, et al. Association between symptom dimensions and categorical diagnoses of psychosis: a cross-sectional and longitudinal investigation. *Schizophr Bull* Jan 2014;40(1):111-119.

**87.** Serpa MH, Ou Y, Schaufelberger MS, et al. Neuroanatomical classification in a population-based sample of psychotic major depression and bipolar I disorder with 1 year of diagnostic stability. *BioMed research international* 2014;2014:706157.

**88.** Angst J, Paksarian D, Cui L, Merikangas KR, Hengartner MP, Ajdacic-Gross V, Rossler W. The epidemiology of common mental disorders from age 20 to 50: results from the prospective Zurich cohort Study. *Epidemiology and psychiatric sciences* Mar 24 2015:1-9.

**89.** Rajkumar RP. Recurrent acute and transient psychotic disorder: A pilot study. *Asian journal of psychiatry* Apr 2015;14:61-64.

**90.** Castagnini AC, Munk-Jorgensen P, Bertelsen A. Short-term course and outcome of acute and transient psychotic disorders: Differences from other types of psychosis with acute onset. *The International journal of social psychiatry* Jun 18 2015.

**91.** Okasha A, el Dawla AS, Khalil AH, Saad A. Presentation of acute psychosis in an Egyptian sample: a transcultural comparison. *Comprehensive psychiatry* Jan-Feb 1993;34(1):4-9.

**92.** Jorgensen P. Comparative outcome of first-admission patients with delusional beliefs. *European psychiatry : the journal of the Association of European Psychiatrists* 1995;10(6):276-281.

**93.** Jorgensen P, Bennedsen B, Christensen J, Hyllested A. Acute and transient psychotic disorder: a 1-year follow-up study. *Acta psychiatrica Scandinavica* Aug 1997;96(2):150-154.

**94.** Das SK, Malhotra S, Basu D. Family study of acute and transient psychotic disorders: comparison with schizophrenia. *Social psychiatry and psychiatric epidemiology* Jun 1999;34(6):328-332.

**95.** Schwartz JE, Fennig S, Tanenberg-Karant M, Carlson G, Craig T, Galambos N, Lavelle J, Bromet EJ. Congruence of diagnoses 2 years after a first-admission diagnosis of psychosis. *Arch Gen Psychiatry* Jun 2000;57(6):593-600.

**96.** Sajith SG, Chandrasekaran R, Sadanandan Unni KE, Sahai A. Acute polymorphic psychotic disorder: diagnostic stability over 3 years. *Acta psychiatrica Scandinavica* Feb 2002;105(2):104-109.

**97.** Jager MDM, Hintermayr M, Bottlender R, Strauss A, Moller HJ. Course and outcome of first-admitted patients with acute and transient psychotic disorders (ICD-10:F23). Focus on relapses and social adjustment. *European archives of psychiatry and clinical neuroscience* Aug 2003;253(4):209-215.

**98.** Jarbin H, von Knorring AL. Diagnostic stability in adolescent onset psychotic disorders. *European child & adolescent psychiatry* Jan 2003;12(1):15-22.

**99.** Amini H, Alaghband-rad J, Omid A, Sharifi V, Davari-Ashtiani R, Momeni F, Aminipour Z. Diagnostic stability in patients with first-episode psychosis. *Australasian psychiatry : bulletin of Royal Australian and New Zealand College of Psychiatrists* Dec 2005;13(4):388-392.

**100.** Correll CU, Lencz T, Smith CW, et al. Prospective study of adolescents with subsyndromal psychosis: characteristics and outcome. *Journal of child and adolescent psychopharmacology* Jun 2005;15(3):418-433.

**101.** Rufino AC, Uchida RR, Vilela JA, Marques JM, Zuardi AW, Del-Ben CM. Stability of the diagnosis of first-episode psychosis made in an emergency setting. *General hospital psychiatry* May-Jun 2005;27(3):189-193.

**102.** Schimmelmann BG, Conus P, Edwards J, McGorry PD, Lambert M. Diagnostic stability 18 months after treatment initiation for first-episode psychosis. *The Journal of clinical psychiatry* Oct 2005;66(10):1239-1246.

**103.** Suda K, Hayashi N, Hiraga M. Predicting features of later development of schizophrenia among patients with acute and transient psychotic disorder. *Psychiatry and clinical neurosciences* Apr 2005;59(2):146-150.

**104.** Whitty P, Clarke M, McTigue O, Browne S, Kamali M, Larkin C, O'Callaghan E. Diagnostic stability four years after a first episode of psychosis. *Psychiatric services* Sep 2005;56(9):1084-1088.

**105.** Abe T, Otsuka K, Kato S. Long-term clinical course of patients with acute polymorphic psychotic disorder without symptoms of schizophrenia. *Psychiatry and clinical neurosciences* Aug 2006;60(4):452-457.

**106.** Addington J, Chaves A, Addington D. Diagnostic stability over one year in first-episode psychosis. *Schizophrenia research* Sep 2006;86(1-3):71-75.

**107.** Thangadurai P, Gopalakrishnan R, Kurian S, Jacob KS. Diagnostic stability and status of acute and transient psychotic disorders. *The British journal of psychiatry : the journal of mental science* Mar 2006;188:293.

**108.** Boks MP, Selten JP, Leask S, Van den Bosch RJ. The 2-year stability of neurological soft signs after a first episode of non-affective psychosis. *European psychiatry : the journal of the Association of European Psychiatrists* Jul 2006;21(5):288-290.

**109.** Rahm C, Cullberg J. Diagnostic stability over 3 years in a total group of first-episode psychosis patients. *Nordic journal of psychiatry* 2007;61(3):189-193.

**110.** Subramaniam M, Pek E, Verma S, Chan YH, Chong SA. Diagnostic stability 2 years after treatment initiation in the early psychosis intervention programme in Singapore. *The Australian and New Zealand journal of psychiatry* Jun 2007;41(6):495-500.

**111.** Fraguas D, de Castro MJ, Medina O, Parellada M, Moreno D, Graell M, Merchan-Naranjo J, Arango C. Does diagnostic classification of early-onset psychosis change over follow-up? *Child psychiatry and human development* Jun 2008;39(2):137-145.

**112.** Haahr U, Friis S, Larsen TK, et al. First-episode psychosis: diagnostic stability over one and two years. *Psychopathology* 2008;41(5):322-329.

**113.** Chang WC, Pang SL, Chung DW, Chan SS. Five-year stability of ICD-10 diagnoses among Chinese patients presented with first-episode psychosis in Hong Kong. *Schizophrenia research* Dec 2009;115(2-3):351-357.

**114.** Crebbin K, Mitford E, Paxton R, Turkington D. First-episode drug-induced psychosis: a medium term follow up study reveals a high-risk group. *Social psychiatry and psychiatric epidemiology* Sep 2009;44(9):710-715.

**115.** Pedros A, Marti J, Gutierrez G, Tenias JM, Ruescas S. [Two-year diagnostic stability and prognosis in acute psychotic episodes]. *Actas espanolas de psiquiatria* Sep-Oct 2009;37(5):245-251.

**116.** Salem MO, Moselhy HF, Attia H, Yousef S. Psychogenic Psychosis Revisited: A Follow up Study. *International journal of health sciences* Jan 2009;3(1):45-49.

**117.** Salvatore P, Baldessarini RJ, Tohen M, Khalsa HM, Sanchez-Toledo JP, Zarate CA, Jr., Vieta E, Maggini C. McLean-Harvard International First-Episode Project: two-year stability of DSM-IV diagnoses in 500 first-episode psychotic disorder patients. *The Journal of clinical psychiatry* Apr 2009;70(4):458-466.

**118.** Aadamsoo K, Saluveer E, Kuunarpuu H, Vasar V, Maron E. Diagnostic stability over 2 years in patients with acute and transient psychotic disorders. *Nordic journal of psychiatry* Dec 2011;65(6):381-388.

**119.** Barak Y, Levy D, Szor H, Aizenberg D. First-onset functional brief psychoses in the elderly. *Canadian geriatrics journal : CGJ* Jun 2011;14(2):30-33.

**120.** Castro-Fornieles J, Baeza I, de la Serna E, et al. Two-year diagnostic stability in early-onset first-episode psychosis. *Journal of child psychology and psychiatry, and allied disciplines* Oct 2011;52(10):1089-1098.

**121.** Kim JS, Baek JH, Choi JS, Lee D, Kwon JS, Hong KS. Diagnostic stability of first-episode psychosis and predictors of diagnostic shift from non-affective psychosis to bipolar disorder: a retrospective evaluation after recurrence. *Psychiatry research* Jun 30 2011;188(1):29-33.

**122.** Moller HJ, Jager M, Riedel M, Obermeier M, Strauss A, Bottlender R. The Munich 15-year follow-up study (MUFUSSAD) on first-hospitalized patients with schizophrenic or affective disorders: assessing courses, types and time stability of diagnostic classification. *European psychiatry : the journal of the Association of European Psychiatrists* May 2011;26(4):231-243.

**123.** Salvatore P, Baldessarini RJ, Tohen M, Khalsa HM, Sanchez-Toledo JP, Zarate CA, Jr., Vieta E, Maggini C. McLean-Harvard International First-Episode Project: two-year stability of ICD-10 diagnoses in 500 first-episode psychotic disorder patients. *The Journal of clinical psychiatry* Feb 2011;72(2):183-193.

**124.** Narayanaswamy JC, Shanmugam VH, Raveendranathan D, Viswanath B, Muralidharan K. Short-term diagnostic stability of acute psychosis: data from a tertiary care psychiatric center in South India. *Indian journal of psychological medicine* Apr 2012;34(2):176-178.

**125.** Pillmann F, Wustmann T, Marneros A. Acute and transient psychotic disorders versus persistent delusional disorders: a comparative longitudinal study. *Psychiatry and clinical neurosciences* Feb 2012;66(1):44-52.

**126.** Kingston T, Scully PJ, Browne DJ, Baldwin PA, Kinsella A, Russell V, O'Callaghan E, Waddington JL. Diagnostic trajectory, interplay and convergence/divergence across all 12 DSM-IV psychotic diagnoses: 6-year follow-up of the Cavan-Monaghan First Episode Psychosis Study (CAMFEPS). *Psychological medicine* Dec 2013;43(12):2523-2533.

**127.** Pope MA, Joober R, Malla AK. Diagnostic stability of first-episode psychotic disorders and persistence of comorbid psychiatric disorders over 1 year. *Canadian journal of psychiatry Revue canadienne de psychiatrie* Oct 2013;58(10):588-594.

**128.** Castagnini A, Foldager L. Epidemiology, course and outcome of acute polymorphic psychotic disorder: implications for ICD-11. *Psychopathology* 2014;47(3):202-206.

**129.** Kapfhammer HP, Reininghaus EZ, Fitz W, Lange P. Clinical course of illness in women with early onset puerperal psychosis: a 12-year follow-up study. *The Journal of clinical psychiatry* Oct 2014;75(10):1096-1104.

**130.** Queirazza F, Semple DM, Lawrie SM. Transition to schizophrenia in acute and transient psychotic disorders. *The British journal of psychiatry : the journal of mental science* 2014;204:299-305.

**131.** Heslin M, Lomas B, Lappin JM, et al. Diagnostic change 10 years after a first episode of psychosis. *Psychological medicine* May 4 2015:1-13.

**132.** Ranjan S, Shakya R, Shyangwa PM. Diagnostic Stability of Acute and Transient Psychotic Disorders in Patients Attending Tertiary Care Hospital. *Journal of Universal College of Medical Sciences* 2015;2(4):7-10.
